# MiR-323a regulates ErbB3/EGFR and blocks gefitinib resistance acquisition in colorectal cancer

**DOI:** 10.1038/s41419-022-04709-9

**Published:** 2022-03-22

**Authors:** Yuanzhou Zhang, Shunshun Liang, Bowen Xiao, Jingying Hu, Yechun Pang, Yuling Liu, Juan Yang, Junpin Ao, Lin Wei, Xiaoying Luo

**Affiliations:** State Key Laboratory of Oncogenes and Related Genes, Shanghai Cancer Institute, Renji Hospital, Shanghai Jiao Tong University School of Medicine, Shanghai, 200032 People’s Republic of China

**Keywords:** Colorectal cancer, Cancer therapeutic resistance

## Abstract

The rapid onset of resistance to epidermal growth factor receptor tyrosine kinase inhibitor (EGFR-TKI) limits its clinical utility in colorectal cancer (CRC) patients, and pan-erb-b2 receptor tyrosine kinase (ErbB) treatment strategy may be the alternative solution. The aim of this study was to develop a possible microRNA multi-ErbB treatment strategy to overcome EGFR-TKI resistance. We detect the receptor tyrosine kinase activity in gefitinib-resistant colorectal cancer cells, ErbB3/EGFR is significantly activated and provides a potential multi-ErbB treatment target. MiR-323a-3p, a tumor suppressor, could target both ErbB3 and EGFR directly. Apoptosis is the miR-323a-3p inducing main biological process by functional enrichment analysis, and The EGFR and ErbB signaling are the miR-323a-3p inducing main pathway by KEGG analysis. MiR-323a-3p promotes CRC cells apoptosis by targeting ErbB3-phosphoinositide 3‐kinases (PI3K)/PKB protein kinase (Akt)/glycogen synthase kinase 3 beta (GSK3β)/EGFR-extracellular regulated MAP kinase (Erk1/2) signaling directly. And miR-323a-3p, as a multi-ErbBs inhibitor, increase gefitinib sensitivity of the primary cell culture from combination miR-323a-3p and gefitinib treated subcutaneous tumors. MiR-323a-3p reverses ErbB3/EGFR signaling activation in gefitinib-resistant CRC cell lines and blocks acquired gefitinib resistance.

## Introduction

Colorectal cancer (CRC) is the second leading cause of cancer-related death worldwide [1–3]. Although screening and excision of precancerous lesions have reduced the mortality of early CRC [4], the overall survival rate is still low due to late CRC metastasis and chemotherapy resistance [5]. In clinical practice, EGFR-targeted therapy (cetuximab) can improve the overall survival (OS) in KRAS wild type colorectal cancer patients [6], which is an important method of targeted therapy for colorectal cancer patients. However, multiple factors, such as APC, KRAS, TP53, BRAF, PIK3CA [7–9], and especially ErbBs activation leading to the heterogeneity of CRC cells, will cause Intrinsic/acquired resistance and limit the therapeutic efficacy of EGFR-TKI in CRC [10].

EGFR-TKI treatment strategies are also limited by high KRAS mutations in CRC [11, 12]. Nevertheless, recent studies have reported that KRAS mutant lung adenocarcinoma resistance to first-generation tyrosine kinase inhibitors (TKIs) is not due to downstream KRAS activation but rather to activation of other ErbB family members [13]. In CRC lines, KRAS G12C inhibition induces higher phospho-ERK rebound than in NSCLC cells and EGFR signaling is the dominant mechanism of CRC resistance to KRAS G12C inhibitors [14]. These results suggest that PAN-ErbB treatment strategies may revert TKI resistance, and EGFR signaling is still a therapeutic target worthy of attention in KRAS mutated CRC.

TKI treatment persistently inhibits EGFR and ErbB2 phosphorylation and persistently inhibits downstream MAPK and JNK signaling [15, 16]. However, phosphorylation of ErbB3 is only transient [17]. ErbB3 plays a key regulatory role in downstream signal transduction of the PI3K/Akt pathway [18, 19] and involves in the mechanisms of widespread acquired resistance to EGFR and ErbB2-targeted therapies in a variety of human malignancies [20–28]. Acquired resistance to cetuximab in CRC is an important clinical problem, and can be attributed to ErbB-2 gene amplification [29]. Strategies to overcome the EGFR-resistance are worth considering by combining with other ErbBs targeted therapies.

MicroRNAs are a class of small endogenous RNAs with a length of about 20–24 nucleotides [30], which play a variety of important regulatory roles in cancer (including inhibition of tumor growth and promotion of apoptosis [31, 32]. The ability to modulate microRNA expression and activity in vivo through microRNA mimics or inhibitors provides an opportunity for the development of innovative therapeutic approaches to cancer [33]. Research on microRNA has moved from the laboratory to the clinical stage, and advances in RNA molecule delivery technology have also made microRNA-based disease treatments more realistic [34]. In this study, we found that ERBB3/EGFR was activated in drug-resistant cell lines. A multi-targeting small RNA, miR-323a-3p, was found to be a ErbBs inhibitor by co-targeting ERBB3/EGFR. MiR-323a-3p inhibiting ERBB3/EGFR led to the deactivation of the PI3K/AKT-Erk1/2 signaling pathway and block acquired resistance to gefitinib in vitro and in vivo. These results provide a possible microRNA multi-ErbB treatment strategy in CRC and a potential way to overcome EGFR-TKI resistance.

## Results

### ErbB3/EGFR is activated in gefitinib-resistant CRC cells

**Fig. 1:**
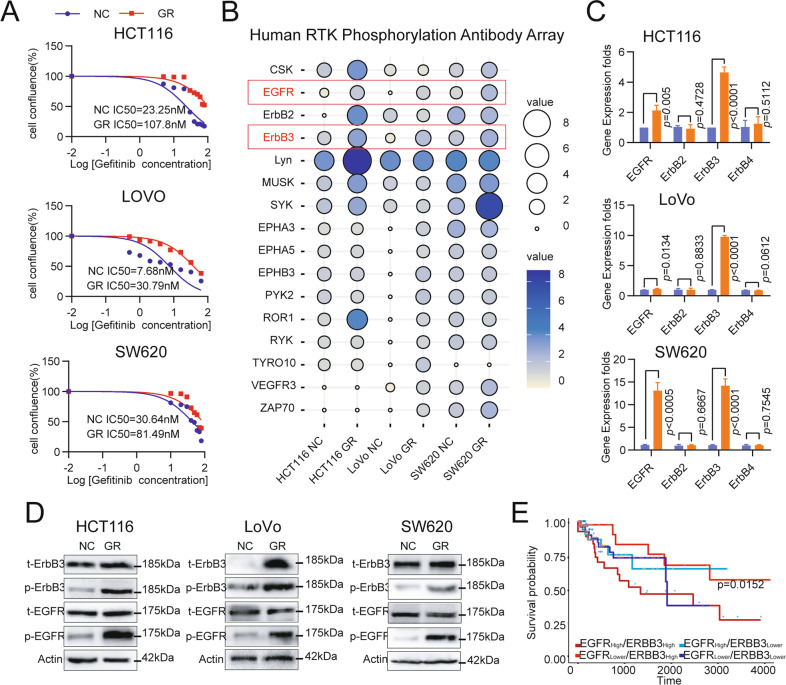
ErbB3 and EGFR are activated in gefitinib-resistant CRC cell lines. **A** HCT116, LoVo, and SW620 cell lines were exposed to increasing concentrations of gefitinib for 48 h to determine the IC50 values by Celigo assay. **B** Detection of Phospho-RTK levels by the Phospho-RTK array in HCT116, LoVo, and SW620 cell lines. Cell was FBS starved for 24 h and stimulated with 20% FBS for ten minutes before harvesting. **C** The ErbBs mRNA levels in HCT116, LoVo, and SW620 cell lines were examined by qRT‐PCR. **D** ErbB3, and EGFR protein expression were examined by western blot assay. E. ErbB3^high^/EGFR^high^ CRC patients are associated with poor prognosis compared to ErbB3^high^/EGFR^low^ group (TCGA datasets).

We constructed CRC gefitinib-resistant cells (HCT116, LoVo, and SW620), the IC50 values were up-regulated to 4.64-fold, 4.01-fold, and 2.66-fold respectively (Fig. 1A). Then, we analyzed the phosphorylation level change of receptor protein tyrosine kinase (RTK) by RTK phosphorylation antibody array. 16 RTKs were activated in gefitinib-resistant HCT116, LoVo, or SW620 cells; and only ErbB3/EGFR were activated in all three gefitinib-resistant cells (Fig. 1B and Supplementary Figure 1A). Furthermore, we detected the ErbBs expression to validate the data of the RTK phosphorylation antibody array. The mRNA levels of EGFR and ErbB3 were increased in all three gefitinib-resistant cells, while ErbB2/ErbB4 were not significantly changed (Fig. 1C). The total and phosphorylated levels of EGFR and ErbB3 protein were upregulated in GR cells (Fig. 1D and SupplementaryFigure 6). We also checked for the possible EGFR T790M mutation during TKI resistance, and no change in mutation level was detected in either the subcutaneous tumor model or in drug-resistant cells (Supplementary Figure 1B and C). ErbB3^high^/EGFR^high^ CRC patients are associated with poor overall survival compared to ErbB3^high^/EGFR^low^ group (TCGA datasets, Fig. 1E). However, there was no significant difference in overall survival between EGFR^High^/ ErbB3^High^ group and EGFR^Low^/ ErbB3^Low^ group. The number of patients for each subset is in Supplementary figure 1F.

### MiR-323a-3p targets ErbB3/EGFR directly and inhibits CRC cell growth

**Fig. 2:**
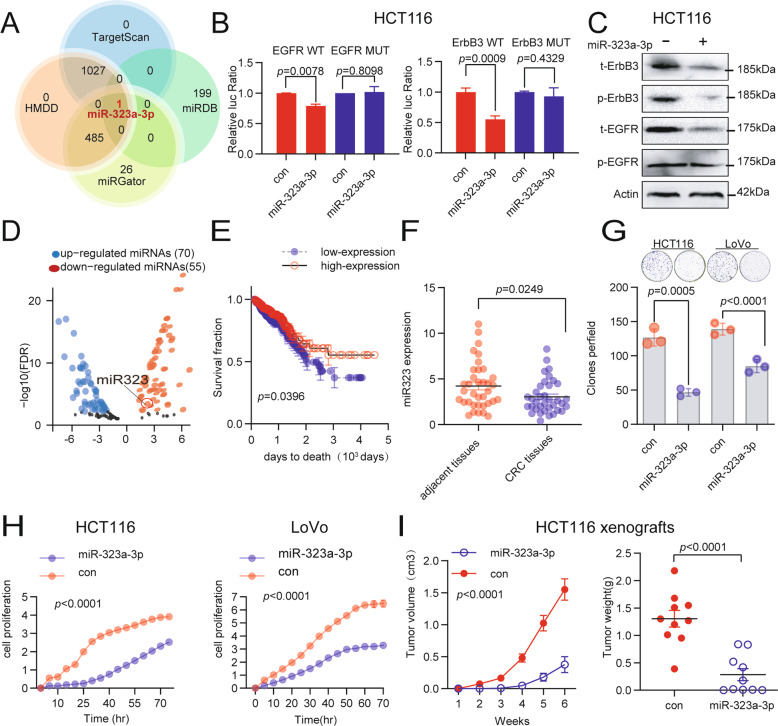
MiR-323a-3p targets ErbB3/EGFR and inhibits CRC cell growth. **A** MiR-323-3p was a candidate microRNA co-targeting ErbB3/EGFR, found in all four microRNA target prediction databases (miRdb, targetScan, HMDD, and miRGator). **B** miR-323-3p inhibited the luciferase activity of the wild type ErbB3/EGFR 3’-UTR contained construct, while did not inhibit the luciferase activity of the mutant ErbB3/EGFR 3’-UTR contained construct. **C** MiR-323a-3p inhibited total and phosphorylated ErbB3/EGFR levels in HCT-116 cells. **D** The expression of miR-323a-3p was significantly downregulated in CRC tissues compared to its normal control (TCGA data). **E** The CRC patients with low miR-323a-3p expression were associated with poor prognosis (TCGA data). **F** Low miR-323a-3p expression was validated in CRC tissues by qRT-PCR assay in CRC tissues (compared to adjacent tissues, *n* = 36). **G** MiR-323a-3p inhibits the clone formation ability in HCT-116 and LoVo cells. **H** MiR-323a-3p inhibits cell growth in HCT-116 and LoVo cells. **I** MiR-323a-3p over-expression inhibits tumor growth in a subcutaneous tumor model (HCT-116 cells, *n* = 6 each group).

To solve the limitations of the current clinical use of TKI-resistance in CRC, we attempted to find microRNAs that can target EGFR/ErbB3 and inhibit the expression of these two genes to improve the efficacy of targeted therapy. Combining analysis in four microRNA target prediction databases (TargetScan, HMDD, miRgator, and miRDB), miR-323a-3p was selected as the candidate microRNA (Fig. 2A, and if we use new prediction tools such as miRWalk, we may get more possibilities for “new” microRNAs to regulate EGFR/ErbB3). Next, a luciferase reporter assay was used to verify that miR-323a-3p directly binds to the 3’UTR of EGFR and ErbB3, and the inhibitory effect of miR-323a-3p on target genes was further verified in HCT116 cell lines. MiR-323a-3p inhibits luciferase activity of EGFR/ErbB3 contained construct, EGFR/ErbB3 protein (Fig. 2B, C, Supplementary Figure 1D and Supplementary Figure 7) and the mRNA level of EGFR/ErbB3 (Supplementary Figure 2A). To determine the biological significance of miR-323a-3p in CRC, we analyzed the miR-323a-3p expression and its relationship with prognosis in the TCGA database. The data showed that miR-323a-3p was downregulated in the CRC tissues (compared to the normal tissue, Fig. 2D), and the CRC patients with low miR-323a-3p expression were associated with poor prognosis, compared to the CRC patients with high miR-323a-3p expression (Fig. 2E). We also found that miR-323a-3p was significantly down-regulated in CRC tissues compared to their adjacent tissues in our cohort (*n* = 36, Fig. 2F). Furthermore, we explored miR-323a-3p function in CRC cells. The results showed that the over-expression of miR-323a-3p in CRC cells (Supplementary Figure 1E) significantly inhibited cell proliferation, migration, and invasion (Fig. 2H and Supplementary Figure 2B-F). We also conducted a clone formation assay and observed that CRC cells transfected with miR-323a-3p formed significantly fewer clones than control cells (Fig. 2G and Supplementary Figure 2G). Subsequently, the over-expression of miR-323a-3p inhibited tumor growth in a subcutaneous tumor model (HCT-116 cells, *n* = 6 each group), the tumor volume inhibition rate was 75%, and the tumor weight inhibition rate was 71% (Fig. 2I and Supplementary Figure 2H).

### MiR-323a-3p inhibits tumor growth by promoting apoptosis in CRC

**Fig. 3:**
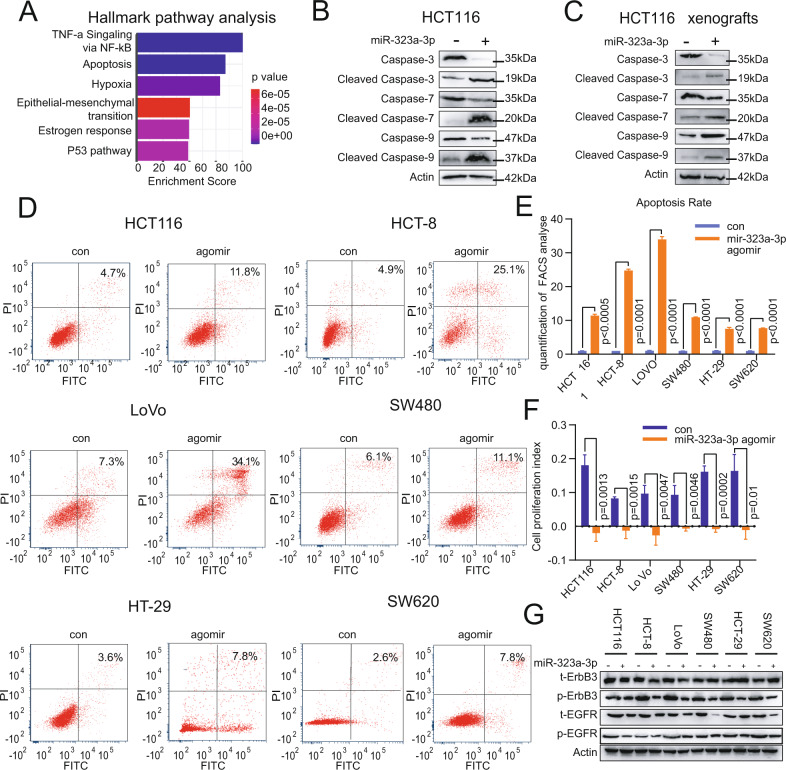
MiR-323a-3p inhibits tumor growth by promoting apoptosis. **A** MiR-323a-3p promoted apoptosis (the second process, hierarchical clustering analysis in miR-323a-3p over-expressing HCT-116 cells). **B** MiR-323a-3p activated apoptotic markers (Caspase 3/7/9, in HCT 116 cells). **C** MiR-323a-3p activated apoptotic markers (Caspase 3/7/9), in miR-323a-3p agomiR treated HCT116 xenograft tissues. **D** MiR-323a-3p promoted apoptosis in CRC cells (miR-323a-3p agomiR treated HCT-116, SW620, LoVo, HCT-8, HT-29 and SW480) for 72 h, by annexin V/PI flow cytometry analysis. **E** The apoptosis rate statistics of miR-323a-3p agomiR treated CRC cells by annexin V/PI flow cytometry analysis. **F** miR-323a-3p agomiR inhibited CRC cell proliferation (miR-323a-3p agomiR treated HCT-116, SW620, LoVo, HCT-8, HT-29 and SW480 for 72 h, by xCELLigence RTCA DP assay). **G** miR-323a-3p agomiR inhibited total and phosphorylated EGFR and ErbB3 protein levels (miR-323a-3p agomiR treated HCT-116, SW620, LoVo, HCT-8, HT-29 and SW480 for 72 h).

To further understand the function of miR-323a-3p, we performed hallmark gene enrichment analysis using RNA-seq data from miR-323a-3p-overexpressing HCT 116 cells. The data revealed that apoptosis was the second enrichment pathway (Fig. 3A). Then, we detected changes in apoptosis-related proteins (caspase3/7/9) in miR-323a-3p over-expression cells. The results showed that the levels of apoptotic markers were activated in HCT116 cells and subcutaneous tumors in response to over-expression of miR-323a-3p (Fig. 3B, C). Similar results were obtained in HCT-8 cells (Supplementary Figure 3B, C). To investigate the apoptotic function of miR-323a-3p in CRC, we selected 6 CRC cell lines treated with miR-323a-3p agomiR (HCT116, LoVo, SW480, SW620, HCT-8 and HT-29) for flow cytometry analysis, and the results showed that miR-323a-3p promoted apoptosis in all 6 types of cells (Fig. 3D, E and Supplementary Figure 8). A similar effect was obtained on the proliferation of the 6 types of cells by RTCA cell growth assay (Fig. 3F and Supplementary Figure 3A). Furthermore, we found that miR-323a-3p could inhibit the total and phosphorylation of ErbB3/EGFR protein levels in agomiR treating six cells (HCT116, LoVo, SW480, SW620, HCT-8 and HT-29, Fig. 3G).

### The molecular regulatory network of miR-323a-3p

**Fig. 4:**
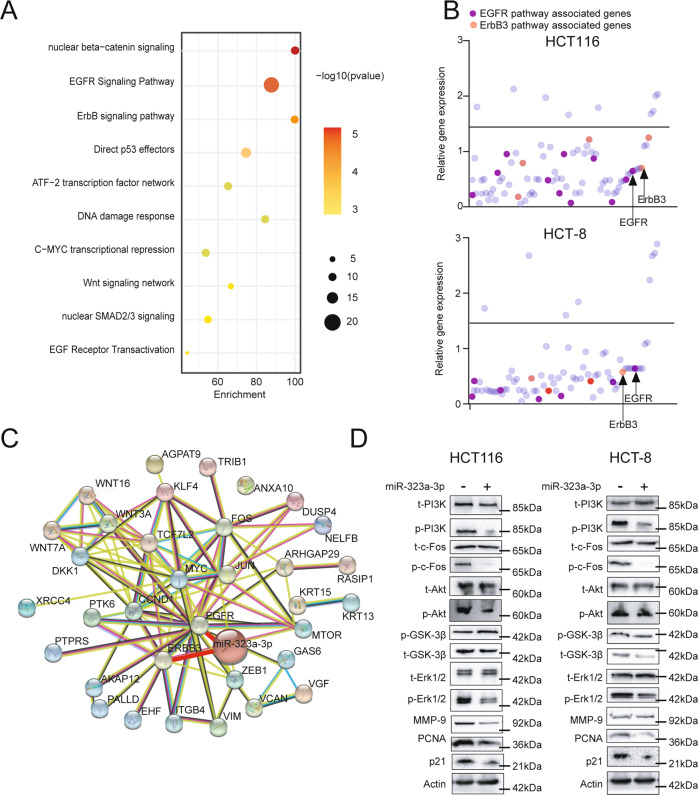
The molecular regulatory network of miR-323a-3p. **A** MiR-323a-3p targeted EGFR and ErbB3 pathway by enriched KEGG pathways analysis. **B** MiR-323a-3p inhibited EGFR and ErbB3 at mRNA level in miR-323a-3p over-expression HCT-116 and HCT-8 cells. We detected the 88 most down-regulated genes in RNA-sequencing data. **C** We constructed the most down-regulated genes protein-protein interaction network by STRING-db, and miR-323a-3p could regulate this network. **D** MiR-323a-3p could inhibit PI3K, c-fos, AKT, GSK3β, and Erk1/2 activation, and inhibit MMP9, PCAN, and p21 expression.

MiR-323a-3p targets ErbB3/EGFR, promotes apoptosis, and inhibits tumor growth in vivo and in vitro. However, its molecular mechanism has not yet been elucidated, so we continued to analyze the RNA-sequencing data of miR-323a-3p-overexpressing HCT116 cells and conducted a pathway analysis by KEGG database (Fig. 4A). The results demonstrated that the deferentially expressed genes were enriched in ErbB3/EGFR-related pathways. There were 88 genes downregulated in the RNA-seq data of miR-323a-3p over-expressed HCT-116 cells compared to the control group. Then, we detected the expression of these genes in over-expressed HCT116 and HCT-8 cells by qRT-PCR assay, and the results showed that EGFR and ErbB3 pathway-associated genes were significantly downregulated (Fig. 4B). Then, we verified the protein level changes of the significantly different genes (PI3K, c-FOS, Akt, GSK-3β, ERK1/2, MMP9, PCNA, and p21) through western blot assay and constructed a protein interaction network of qRT-PCR assay validated deferentially expressed genes using String-db (Fig. 4C, D and Supplementary Fig. 9). In addition, EGFR/ErbB3 agonist (NSC228155) rescued phosphorylation of ErbB3/EGFR and apoptosis effect by miR-323a-3p (Supplementary Fig. 4). These results reveal that miR-323a-3p could inhibit phosphorylation of PI3K/Akt/GSK3β-Erk1/2, downstream of ErbB3/EGFR pathway, by targeting ErbB3/EGFR.

### MiR-323a-3p reverse ErbB3/EGFR activation in gefitinib-resistant CRC cell lines

**Fig. 5:**
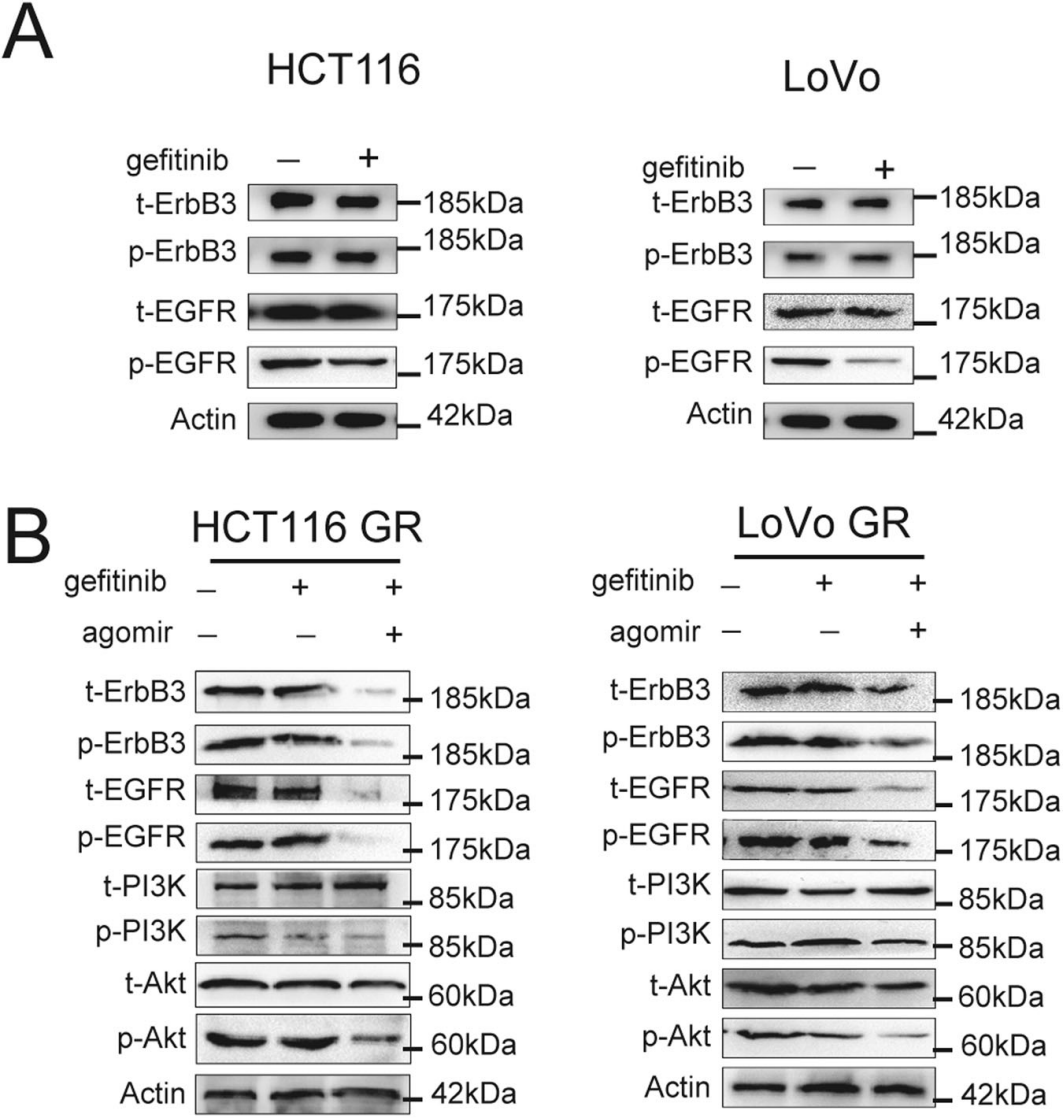
MiR-323a-3p reverse ErbB3/EGFR activation in gefitinib-resistant CRC cell lines. **A** Gefitinib inhibited total and phosphorylated levels of ErbB3/EGFR in HCT116 and LoVo cells. **B** Gefitinib and miR-323a-3p inhibited total and phosphorylated levels of ErbB3/EGFR in gefitinib-resistant HCT-116 and LoVo cells, while Gefitinib did not inhibit alone. Gefitinib and miR-323a-3p inhibited phosphorylated levels of PI3K/AKT (downstream of ErbB3) activation.

Gefitinib significantly inhibited the phosphorylation of EGFR but weakly affected on phosphorylation of ErbB3 in HCT116 and LoVo cells (Fig. 5A and Supplementary Figure 10). Both phosphorylation levels of ErbB3 and EGFR protein in drug-resistant cell lines had no significant change in response to treatment with 200 nM gefitinib for 48 h (Fig. 5B and Supplementary Figure 11). However, the phosphorylation levels of ErbB3/EGFR protein were significantly reduced after adding MiR-323a-3p agomiR for 72 h (Fig. 5B and Supplementary Figure 3D). And gefitinib also has no effect on PI3K/Akt phosphorylation alone in resistance cells. However, combination gefitinib with miR-323a-3p agomiR could inhibit PI3K/Akt phosphorylation in these cells (Fig. 5B).

### MiR-323a-3p blocks acquired gefitinib resistance in a xenograft model

**Fig. 6:**
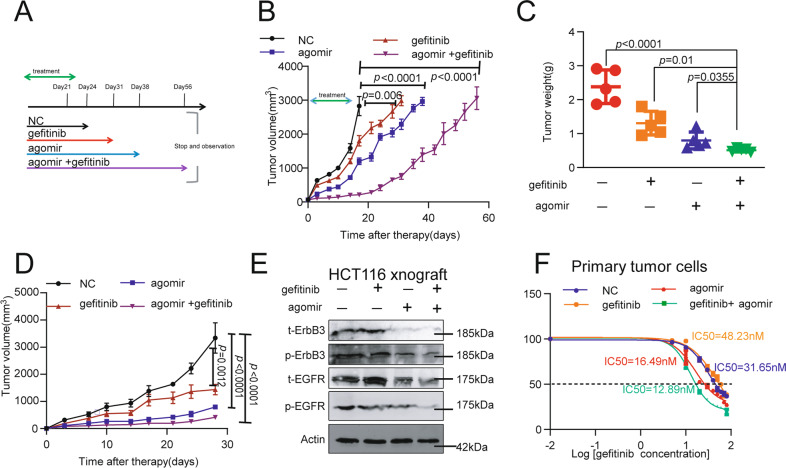
MiR-323a-3p blocks acquired gefitinib resistance in a xenograft model. **A** Schematic diagram of the treatment model. **B** MiR-323a-3p inhibited subcutaneous tumors growth, combined with gefitinib (*n* = 7). **C** MiR-323a-3p inhibited subcutaneous tumors weight, combined with gefitinib (*n* = 5). **D** MiR-323a-3p inhibited subcutaneous tumors volume, combined with gefitinib (*n* = 5). **E** MiR-323a-3p inhibited total and phosphorylated levels of EGFR and ErbB3 in subcutaneous tumor tissue samples, combined with gefitinib (*n* = 5). **F** MiR-323a-3p blocked acquired gefitinib resistance in a xenograft model, the IC50 value was upregulated to 48.23 nM from 31.65 nM in the primary culture from gefitinib treated subcutaneous tumors, and the IC50 value was down-regulated to 12.89 nM in the primary culture from miR-323a-3p/gefitinib co-treated subcutaneous tumors.

MiR-323a-3p combined with gefitinib inhibit tumor growth of subcutaneous HCT116 cells (Fig. 6A-B and Supplementary Figure 5A, B). After combined administration of miR-323a-3p and gefitinib, the subcutaneous tumors composed of HCT116 cells maintained a period of growth arrest, and the growth rate of subcutaneous tumors is much lower than the monotherapy group. Additionally, the HCT116 subcutaneous tumor model showed similar results in tumor weight and volume four weeks after administration (Fig. 6C, D and Supplementary Figure 5C), and the tumor weight and volume were slightly decreased after administration of miR-323a-3p or gefitinib, with tumor growth inhibition rates of 27% and 33%, respectively. After combination administration, tumor weight (inhibition rates of 78.4%) and volume (inhibition rates of 87.7%) were smaller than those of the single administration group. Gefitinib did not significantly change the phosphorylated ErbB3/EGFR level. MiR-323a-3p suppressed ErbB3/EGFR phosphorylation at a certain level alone (Fig. 6E and Supplementary Figure 12). Combination administration of miR-323a-3p with gefitinib further inhibits the ErbB3/EGFR phosphorylation. We detected the IC50 value of the primary cells from four groups subcutaneous tumors. More meaningfully, the IC50 values of the combination group were 59% down-regulated compared to the control group. Otherwise, the IC50 value of the gefitinib group was increased by 52% compared to the control group. And the IC50 value of the miR-323a-3p group exhibited 47% inhibition compared to the control group (Fig. 6F and Supplementary Figure 5D, E).

## Discussion

**Fig. 7:**
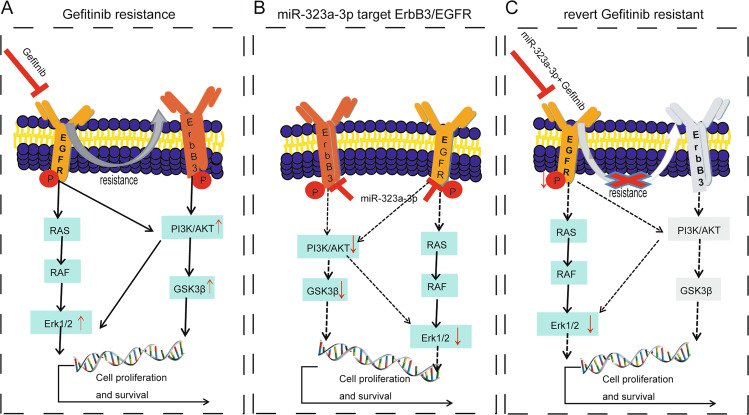
MiR-323a-3p inhibits gefitinib resistance acquisition by targeting ErbB3/PI3K/Akt-EGFR/Erk1/2 signaling in colorectal cancer. **A** Erbb3/EGFR pathway is activated in gefitinib resistance CRC cells. **B** MiR-323a-3p inhibits phosphorylation of Erk1/2-PI3K/Akt/GSK3β by targeting ErbB3/EGFR directly. **C** MiR-323a-3p blocks acquired gefitinib resistance by targeting ErbB3/PI3K/Akt-EGFR/Erk1/2 signaling.

The EGFR-TKI treatment strategy is the current clinical treatment approach, although it still faces limitations. Gefitinib is the first generation of EGFR-TKI targeting EGFR-19del and EGFR-L858R mutations [35, 36], but its efficacy in CRC is lower than that of other types of cancer [37, 38]. Compared to its efficacy in the treatment of non-small cell lung cancer, gefitinib in the phase I trial in CRC patients achieved stable disease without objective response in terms of tumor size [39, 40]. Although monoclonal EGFR-TKIs, such as cetuximab, are commonly used in the clinical treatment of CRC [41], there are therapeutic limitations and these drugs are ineffective in patients with RAS gene mutations [42–44]. Recent reports have shown that the addition of cetuximab to perioperative chemotherapy significantly reduces progression-free survival in patients with resectable liver metastases from colorectal cancer [45, 46]. In addition, EGFR inhibition initiates a mechanism of rapid resistance involving non-EGFR ErbB family members [13]. This mechanism of tumor escape illuminates the current disappointing EGFR-TKI results and suggests the high therapeutic potential of pan-ErbB inhibitors. In this study, we found that ErbB3/EGFR activated in CRC gefitinib-resistant cell lines (Fig. 7A), suggesting multi-targeted therapy of EGFR and ErbB3 could solve the TKI-resistance issue.

ErbB3 is often over-expressed in CRC [47, 48] and plays a key regulatory role in downstream signal transduction of the PI3K/Akt pathway [49]. ErbB3 over-expression and the consequent activation of PI3K/Akt signaling lead to the development of resistance to tyrosine kinase inhibitors such as gefitinib in ErbB2 over-expressed breast cancers [50]. More notably, the absence of ErbB3 expression in poorly differentiated colorectal cancer cells enhances gefitinib sensitivity [51]. Because ErbB3 provides a heterodimerization platform for other members of the EGFR family [52], it is involved in the mechanism of widespread acquired resistance to targeted therapies for EGFR and ErbB2 in a variety of human malignancies [53–55]. Therefore, we believe that ErbB3/EGFR inhibition is a promising treatment strategy in CRC. In this study, we develop a strategy to co-target ErbB3/EGFR by miR-323a-3p and block the acquired TKI-resistance.

MiR-323a-3p has shown a variety of regulatory functions in the existing studies. For example, miR-323a-3p inhibits ErbB4 and affects depression [56], inhibits UHMK1 to down-regulate tumor growth in colorectal cancer [57], and inhibits PUM1/eIF2 axis in breast cancer [58]. In this study, miR-323a-3p inhibits the downstream PI3K/AKT/GEK3β-Erk1/2 pathway by targeting EGFR/ ErbB3 then blocks TKI-resistance in CRC. And Luciferase assay results showed a weaker effect, suggesting that the interaction of miR-323a-3p with the 3’UTR may be just one way of its regulation of EGFR/ ErbB3.

In summary, our findings demonstrated ErbB3/EGFR activated in gefitinib-resistance CRC cells. MiR-323a-3p blocks TKI-resistance by targeting ErbB3/EGFR. These findings provide a possible microRNA Pan-ErbB treatment strategy, and potential combination therapy to block acquired resistance.

## Materials and methods

### Cell cultures and reagents

HCT116, LoVo, SW620, SW480, HT-29, and HCT-8 human CRC cell lines were purchased from the American Type Culture Collection (ATCC, Rockville, MD, USA). HCT116/HT-29 cells were preserved in McCoy’s 5 A medium, HCT-8 cells were preserved in 1640 medium, LoVo cells were preserved in F-12K medium (Kaighn’s Modification of Ham’s F-12 Medium), and SW480/SW620 cells were preserved in Leibovitz’s L-15 medium. The media were supplemented with 10% fetal bovine serum, and cells were incubated in a humidified atmosphere of 95% air and 5% CO2 at 37 °C.

HCT116 GR (gefitinib-resistant), LoVo GR, SW620 GR, and HT-29 GR cells in the logarithmic growth phase (convergence degree 60%–80%) were added with gefitinib (ZD1839) with an initial concentration of 1/10 of the parent cell line IC50 for 24 h. The culture medium was discarded, washed twice with PBS, and the drug-free medium was replaced. After cell growth resumed, the cells were treated with low concentration for 24 h. After the cells proliferate to normal morphology, the above drug shock was repeated, with 6–8 times of shock at each concentration. After the cells grew stably at this concentration, the culture was continued by increasing the concentration of drugs by 1.2–1.5 times. Drug induction lasted for 6 months until the cells were able to grow steadily in the concentration of the drug.

### Transfections

Lentiviruses containing miR-323a or control were purchased from GeneChem (Shanghai, China), and the transfection of lentivirus into CRC cells was performed according to the manufacturer’s protocol. After lentivirus infection, monoclonal cells were selected and cultured to check for expression of miR-323a-3p/5p by qRT-PCR. MicroRNA mimics were synthesized by RiboBio (Guangzhou, China) and transfected into CRC cells using Lipofectamine™ 2000 (Invitrogen, USA) according to the manufacturer’s protocol.

### Receptor tyrosine kinase phosphorylation profile

To investigate the activation/phosphorylation of RTKs, we used the Human RTK Phosphorylation Antibody Array Membrane (ab193662, Abcam). The human phospho-RTK antibody array is a nitrocellulose membrane with 71 different anti-RTK antibodies spotted in duplicate on it, including 4 positive and 3 negative controls and 1 blank.

To perform a proteome profiler array experiment, cell lysates were prepared from GR or NC cells using Cell Lysis Buffer supplemented with Phosphatase Inhibitor and Protease Inhibitor Cocktail and stored at −80 °C until use. For each cell lysate, 200 μg of total protein (determined by the Pierce BCA Protein Assay (Fisher Scientific) was diluted 1:5 with blocking buffer, placed onto each membrane, and incubated overnight at 4 °C (16 h). The antibody array membranes were washed and subsequently incubated with a biotinylated anti-phosphotyrosine antibody overnight at 4 °C to detect phosphorylated tyrosine on activated receptors. After washing and incubation with HRP-streptavidin, the membranes were visualized using a chemiluminescence-based detection method.

### Cell viability assay

Tumor cells (2–3 × 10^3^ cells/100 μL/well) in media supplemented with 10% FBS were plated in 96-well plates and cultured with the indicated compound for 72 h. After culturing, cell viability was measured using a CCK-8 kit (Dojindo Laboratories). The percentage of growth was determined relative to the untreated controls. Experiments were repeated at least three times with triplicate samples.

#### Patients and bioinformatics analysis

Paired colorectal tumor tissues and their corresponding adjacent nontumor colorectal tissues (5 cm away from the lesions) were collected from patients who underwent curative surgery for CRC at Renji Hospital, Shanghai, China. A CRC diagnosis was confirmed by histological examination, and the relevant clinical and pathological information was retrieved from the hospital database. The expression of microRNAs in CRC and normal controls was determined using the TCGA database, and a heat map and volcano map were acquired using R.

#### RNA extraction and quantitative real-time RT-PCR (qRT-PCR)

Total RNA was extracted from cell lines and frozen tumor samples using TRIzol reagent (Invitrogen, Carlsbad, CA). cDNA was reverse-transcribed from 1 µg of RNA using an SYBR® Prime ScriptTM RT-PCR kit (Takara Biochemicals, Tokyo, Japan), and quantitative PCR was performed using SYBR Select Master Mix (Roche, Switzerland) and gene-specific primers on an ABI PRISM® 7500HT Real-Time PCR System. The thermal cycling conditions were as follows: an initial step at 95 °C for 15 s followed by 40 cycles of 95 °C for 5 s and 60 °C for 30 s. Each experiment was performed in a 20-µl reaction volume containing 10 µl of SYBR® Prime Ex TaqTM II (2×), 1 µl of forward primer and reverse primer (10 µM each), 2 µl of cDNA, and 7 µl of H2O. β-Actin was used as an internal control. The quantification of the mRNA was calculated using the comparative Ct (the threshold cycle) method according to the following formula: Ratio = 2^−∆∆CT^ = 2^−[∆Ct(sample)-∆Ct(calibrator)]^, where ∆Ct is equal to the Ct of the target gene minus the Ct of the endogenous control gene (β-actin). The primers were as follows: EGFR (F: 5′-CCAAGGCACGAGTAACAAGC-3′; R: 5′- TCCCAAGGACCACCTCACAG-3′); ErbB3 (F: 5′-GGTGATGGGGAACCTTGAGAT-3′; R: 5′-CTGTCACTTCTCGAATCCACTG-3′).

### EGFR T790M mutation detection

We purchased all probe qPCR mix (or UNG) reagents from Takara (Tokyo, Japan) and custom ordered primers and probes from GENEWIZ (Guangzhou, China). We performed probe qPCR analysis on an ABI PRISM®7500HT Real-Time PCR System as previously described under the following PCR conditions: EGFR T790M: forward primer, 5′-GCCTGCTGGGCATCTG-3′, reverse primer, 5′-TCTTTGTGTTCCCGGACATAGTC-3′. The probe sequences were 5′-VIC-ATGAGCTGCGTGATGAG-MGB-NFQ-3′ and 5′-FAM-ATGAGCTGCATGATGAG-MGB-NFQ-3′. Cycling conditions: 95 °C × 10 min (1 cycle), 40 cycles of 94 °C × 30 s and 58 °C × 1 min, followed by a 10 °C hold.

### Antibodies and western blotting

Protein aliquots of 25 μg each were resolved by SDS polyacrylamide gel electrophoresis (Bio-Rad, Hercules, CA) with the appropriate antibodies. Electrophoresed protein samples were transferred to polyvinylidene difluoride membranes (Bio-Rad). After being washed three times, the membranes were incubated in blotting-grade blocker (Bio-Rad) for 1 h at room temperature and overnight at 4 °C with primary antibodies to t-EGFR, p-EGFR, t-ErbB3, p-ErbB3, t-PI3K, p-PI3K, p-Akt, t-Akt, t-Erk1/2, p-Erk1/2, t-GSK3β,p-GSK3β, MMP9, PCNA,p21, Caspase-3, Cleaved-Caspase-3, Caspase-7, Cleaved-Caspase-7, Caspase-9, Cleaved-Caspase-9, and β-actin (1:1,000 dilution; Supplementary Table 1). After being washed three times, the membranes were incubated for 1 h at room temperature with an HRP-conjugated species-specific secondary antibody. Immunoreactive bands were visualized using SuperSignal West Dura Extended Duration Substrate Enhanced Chemiluminescent Substrate (Pierce Biotechnology). Each experiment was independently performed at least three times.

### Cell proliferation

The RTCA xCELLigence system (ACEA Biosciences Inc., The Netherlands) was used to measure cell proliferation in real-time. CRC cells were placed at a density of 4000–8000/well, and E-plates were then transferred to the RTCA instrument for automated real-time monitoring under standard incubator conditions. Cell proliferation was monitored every 30 min. After 72 h, the measurement was stopped, and the results were analyzed using RTCA software and the results were analyzed after an additional 24 h.

### Colony formation assay

Transfected CRC cells were seeded into 6-well plates at a density of 200–800 cells per well and incubated for 2 weeks. The cells were fixed and stained in a dye solution containing 0.1% crystal violet and 100% methanol. The number of colonies was subsequently counted and analyzed.

### Flow cytometry

Apoptosis was determined by fluorescence-activated cell sorting (FACS) flow cytometry. The transfected cells collected after trypsinization were washed twice with PBS and resuspended in 1X binding buffer. Cells were then stained using 10 μL FITC-labeled buffer for 20 min and 10 μL PE-labeled buffer for 5 min according to the manufacturer’s instructions. The apoptosis rate was analyzed by FACS flow cytometry (BD Biosciences, Heidelberg, Germany).

### Luciferase reporter assay

HCT116 cells grown in 24-well plates were co-transfected with luciferase reporter (200 ng per well), miR-323a-3p-mimic (200 ng per well), and 10 ng *Renilla* using Lipofectamine™ 2000. Forty-eight hours later, a Dual-Luciferase Reporter Assay kit (Promega, USA) was used to measure the luciferase and Renilla activities according to the manufacturer’s instructions. The relative luciferase activity was determined using BioTek Synergy 2 (BioTek, USA), and the transfection efficiency was normalized to Renilla activity.

### CDX models

Suspensions of 3 × 10^6^ cells were injected subcutaneously into the flanks of four-week-old nude mice (Each group contained at least 5 mice). Once the mean tumor volume reached ~50–100 mm^3^, the mice were orally administered gefitinib and injected with agomiR three times per week. Tumors were measured three times per week using calipers, and tumor volumes were calculated as width^2^ × length/2. Mice were euthanized after 28 days, tumors were collected and weighed, and total RNA and protein were prepared from tumor tissues for qRT-PCR and western blot analysis.

### Statistical analysis

All the data were obtained from experiments with adequate sample sizes and presented as the mean ± standard deviation (SD). Statistical analysis was performed using Prism 8 (GraphPad, San Diego, CA, USA). Two-way ANOVA, one-way ANOVA, χ2 tests, and the two-tailed unpaired Student’s *t*-tests were used to assess the significance of the differences between groups. The Kaplan–Meier method and log-rank tests of survival were used to study the role of miR-323a-3p. Each treatment was analyzed in at least three experiments.

## Supplementary information

SUPPLEMENTAL MATERIAL

SUPPLEMENT rna seq TABLE

aj-checklist

## Data Availability

The raw/processed data required to reproduce these findings cannot be shared at this time as the data also forms part of an ongoing study.

## References

[1] Jemal A, Bray F, Center MM, Ferlay J, Ward E, Forman D (2011). Global cancer statistics. CA: a cancer J clinicians.

[2] Arnold M, Sierra MS, Laversanne M, Soerjomataram I, Jemal A, Bray F. Global patterns and trends in colorectal cancer incidence and mortality. Gut. 2016;66:683–91. 10.1136/gutjnl-2015-310912 26818619

[3] Zhao S, Mi Y, Guan B, Zheng B, Wei P, Gu Y (2020). Tumor-derived exosomal miR-934 induces macrophage M2 polarization to promote liver metastasis of colorectal cancer. J Hematol Oncol.

[4] Tang X, Sun G, He Q, Wang C, Shi J, Gao L (2020). Circular noncoding RNA circMBOAT2 is a novel tumor marker and regulates proliferation/migration by sponging miR-519d-3p in colorectal cancer. Cell Death Dis.

[5] Du F, Cao T, Xie H, Li T, Sun L, Liu H (2020). KRAS Mutation-Responsive miR-139-5p inhibits Colorectal Cancer Progression and is repressed by Wnt Signaling. Theranostics.

[6] Byrne M, Saif MW (2019). Selecting treatment options in refractory metastatic colorectal cancer. OncoTargets Ther.

[7] Yaeger R, Chatila WK, Lipsyc MD, Hechtman JF, Cercek A, Sanchez-Vega F (2018). Clinical Sequencing Defines the Genomic Landscape of Metastatic Colorectal Cancer. Cancer Cell.

[8] Bronte G, Cicero G, Cusenza S, Galvano A, Musso E, Rizzo S (2013). Monoclonal antibodies in gastrointestinal cancers. Expert Opin Biol Ther.

[9] Knight JRP, Alexandrou C, Skalka GL, Vlahov N, Pennel K, Officer L, et al. MNK inhibition sensitizes KRAS-mutant colorectal cancer to mTORC1 inhibition by reducing eIF4E phosphorylation and c-MYC expression. Cancer Discov. 2020;11:1228–47. 10.1158/2159-8290.CD-20-0652 PMC7611341 33328217

[10] Mésange P, Bouygues A, Ferrand N, Sabbah M, Escargueil AE, Savina A, et al. Combinations of Bevacizumab and Erlotinib show activity in colorectal cancer independent of RAS status. Clin Cancer Res. 2018. 24:2548–58. 10.1158/1078-0432.CCR-17-3187 29490990

[11] Canon J, Rex K, Saiki AY, Mohr C, Cooke K, Bagal D, et al. The clinical KRAS(G12C) inhibitor AMG 510 drives anti-tumour immunity. Nature. 2019. 575:217–23. 10.1038/s41586-019-1694-1 31666701

[12] Christensen JG, Hallin J, Engstrom LD, Hargis L, Calinisan A, Aranda R, et al. The KRASG12C Inhibitor, MRTX849, Provides Insight Toward Therapeutic Susceptibility of KRAS Mutant Cancers in Mouse Models and Patients. Cancer Discov. 2019;10:54–71. 10.1158/2159-8290.CD-19-1167 PMC6954325 31658955

[13] Moll HP, Pranz K, Musteanu M, Grabner B, Hruschka N, Mohrherr J, et al. Afatinib restrains K-RAS-driven lung tumorigenesis. Sci Transl Med. 2018;10:eaao2301. 10.1126/scitranslmed.aao2301 PMC7610658 29925635

[14] Amodio V, Yaeger R, Arcella P, Cancelliere C, Lamba S, Lorenzato A, et al. EGFR blockade reverts resistance to KRAS G12C inhibition in colorectal cancer. Cancer Discov. 2020;10:1129–39. 10.1158/2159-8290.CD-20-0187 PMC7416460 32430388

[15] Conlon NT, Kooijman JJ, van Gerwen SJC, Mulder WR, Zaman GJR, Diala I, et al. Comparative analysis of drug response and gene profiling of HER2-targeted tyrosine kinase inhibitors. Br J Cancer. 2021;124:1249–59. 10.1038/s41416-020-01257-x PMC8007737 33473169

[16] Hsu CC, Liao BC, Liao WY, Markovets A, Stetson D, Thress K, et al. Exon-16-skipping HER2 as a novel mechanism of osimertinib-resistance in EGFR L858R/T790M-positive non-small-cell lung cancer. J Thorac Oncol. 2019;15:50–61. 10.1016/j.jtho.2019.09.006 31557536

[17] Yonesaka K, Tanaka K, Kitano M, Kawakami H, Hayashi H, Takeda M (2019). Aberrant HER3 ligand heregulin-expressing head and neck squamous cell carcinoma is resistant to anti-EGFR antibody cetuximab, but not second-generation EGFR-TKI. Oncogenesis.

[18] Loree JM, Bailey AM, Johnson AM, Yu Y, Wu W, Bristow CA, et al. Molecular Landscape of ERBB2/ERBB3 Mutated Colorectal Cancer. J Natl Cancer Inst. 2018;110:1409–17. 10.1093/jnci/djy067 PMC6292791 29718453

[19] Berger MD, Stintzing S, Heinemann V, Cao S, Yang D, Miyamoto Y (2018). Genetic variations within the HER3 gene predict outcome for mCRC patients treated with first-line FOLFIRI/bevacizumab or FOLFIRI/cetuximab: Data from FIRE-3. Ann Oncol.

[20] Yakes FM, Chinratanalab W, Ritter CA, King W, Seelig S, Arteaga CL (2002). Herceptin-induced inhibition of phosphatidylinositol-3 kinase and Akt Is required for antibody-mediated effects on p27, cyclin D1, and antitumor action. Cancer Res.

[21] Claus J, Patel G, Ng T, Parker PJ (2014). A role for the pseudokinase HER3 in the acquired resistance against EGFR- and HER2-directed targeted therapy. Biochemical Soc Trans.

[22] Capparelli C, Purwin TJ, Heilman SA, Chervoneva I, McCue PA, Berger AC (2018). ErbB3 Targeting Enhances the Effects of MEK Inhibitor in Wild-Type BRAF/NRAS Melanoma. Cancer Res.

[23] Kugel CH, Hartsough EJ, Davies MA, Setiady YY, Aplin AE (2014). Function-blocking ERBB3 antibody inhibits the adaptive response to RAF inhibitor. Cancer Res.

[24] Abel EV, Basile KJ, Kugel CH, Witkiewicz AK, Le K, Amaravadi RK (2013). Melanoma adapts to RAF/MEK inhibitors through FOXD3-mediated upregulation of ERBB3. The. J Clin Investig.

[25] Noto A, De Vitis C, Roscilli G, Fattore L, Malpicci D, Marra E (2013). Combination therapy with anti-ErbB3 monoclonal antibodies and EGFR TKIs potently inhibits non-small cell lung cancer. Oncotarget..

[26] Fattore L, Marra E, Pisanu ME, Noto A, de Vitis C, Belleudi F (2013). Activation of an early feedback survival loop involving phospho-ErbB3 is a general response of melanoma cells to RAF/MEK inhibition and is abrogated by anti-ErbB3 antibodies. J Transl Med.

[27] Ruggiero CF, Malpicci D, Fattore L, Madonna G, Vanella V, Mallardo D, et al. ErbB3 Phosphorylation as Central Event in Adaptive Resistance to Targeted Therapy in Metastatic Melanoma: Early Detection in CTCs during Therapy and Insights into Regulation by Autocrine Neuregulin. Cancers. 2019;11:1425. 10.3390/cancers11101425 PMC6826737 31557826

[28] Bosch-Vilaro A, Jacobs B, Pomella V, Abbasi Asbagh L, Kirkland R, Michel J (2017). Feedback activation of HER3 attenuates response to EGFR inhibitors in colon cancer cells. Oncotarget..

[29] Han Y, Peng Y, Fu Y, Cai C, Guo C, Liu S (2020). MLH1 Deficiency Induces Cetuximab Resistance in Colon Cancer via Her-2/PI3K/AKT Signaling. Adv Sci.

[30] Singh R, Ha SE, Wei L, Jin B, Zogg H, Poudrier SM, et al. MiR-10b-5p Rescues Diabetes and Gastrointestinal Dysmotility. Gastroenterology. 2021;160:1662–78.e18. 10.1053/j.gastro.2020.12.062 PMC8532043 33421511

[31] Borchardt H, Ewe A, Morawski M, Weirauch U, Aigner A. miR24-3p activity after delivery into pancreatic carcinoma cell lines exerts profound tumor-inhibitory effects through distinct pathways of apoptosis and autophagy induction. Cancer Lett. 2021;503:174–84. 10.1016/j.canlet.2021.01.018 33508384

[32] Zhang C, Zhu Z, Gao J, Yang L, Dang E, Fang H, et al. Plasma exosomal miR-375-3p regulates mitochondria-dependent keratinocyte apoptosis by targeting XIAP in severe drug-induced skin reactions. Sci Transl Med. 2020;12:eaaw6142. 10.1126/scitranslmed.aaw6142 33328332

[33] Wyss CB, Duffey N, Peyvandi S, Barras D, Martinez Usatorre A, Coquoz O (2021). Gain of HIF1 Activity and Loss of miRNA let-7d Promote Breast Cancer Metastasis to the Brain via the PDGF/PDGFR Axis. Cancer Res.

[34] Mirna M, Paar V, Topf A, Kraus T, Sotlar PK, Aigner PA, et al. A new player in the game: treatment with antagomiR-21a-5p significantly attenuates histological and echocardiographic effects of experimental autoimmune myocarditis. Cardiovasc Res. 20211;118:556–72. 10.1093/cvr/cvab015 33483746

[35] Cho JH, Lim SH, An HJ, Kim KH, Park KU, Kang EJ, et al. Osimertinib for Patients With Non-Small-Cell Lung Cancer Harboring Uncommon EGFR Mutations: A Multicenter, Open-Label, Phase II Trial (KCSG-LU15-09). J Clin Oncol. 2019;38:488–95. 10.1200/JCO.19.00931 PMC7098834 31825714

[36] Huang TY, Chang TC, Chin YT, Pan YS, Chang WJ, Liu FC, et al. NDAT Targets PI3K-Mediated PD-L1 Upregulation to Reduce Proliferation in Gefitinib-Resistant Colorectal Cancer. Cells. 2020;9:1830. 10.3390/cells9081830 PMC7464180 32756527

[37] Blanke CD (2005). Gefitinib in colorectal cancer: if wishes were horses. J Clin Oncol.

[38] Ono M, Kuwano M (2006). Molecular mechanisms of epidermal growth factor receptor (EGFR) activation and response to gefitinib and other EGFR-targeting drugs. Clin Cancer Res..

[39] Yang Z, Hackshaw A, Feng Q, Fu X, Zhang Y, Mao C, et al. Comparison of gefitinib, erlotinib and afatinib in non-small cell lung cancer: A meta-analysis. Int J Cancer. 2017;140:2805–19. 10.1002/ijc.30691 28295308

[40] Hartmann JT, Kroening H, Bokemeyer C, Holtmann M, Schmoll HJ, Kanz L (2005). Phase I study of gefitinib in combination with oxaliplatin and weekly 5-FU/FA (FUFOX) for second-/third-line treatment in patients (pts) with metastatic colorectal cancer (CRC). J Clin Oncol.

[41] Tabernero J, Grothey A, Van Cutsem E, Yaeger R, Wasan H, Yoshino T (2021). Encorafenib Plus Cetuximab as a New Standard of Care for Previously Treated BRAF V600E-Mutant Metastatic Colorectal Cancer: Updated Survival Results and Subgroup Analyses from the BEACON Study. J Clin Oncol.

[42] Ho CS, Cheng AC, Li L, Ho WM, Hui EP, To KF (2018). A pilot case-control study of second or third-line treatment with cetuximab-containing chemotherapy (cetux-chemo) in patients (pts) with metastatic colorectal cancer (mCRC) who were previously treated with cetux-chemo. Ann Oncol..

[43] Kopetz S, Guthrie KA, Morris VK, Lenz HJ, Magliocco AM, Maru D, et al. Randomized Trial of Irinotecan and Cetuximab With or Without Vemurafenib in BRAF-Mutant Metastatic Colorectal Cancer (SWOG S1406). J Clin Oncol. 2021;39:285–94. 10.1200/JCO.20.01994 PMC8462593 33356422

[44] Kopetz S, Grothey A, Yaeger R, Van Cutsem E, Desai J, Yoshino T, et al. Encorafenib, Binimetinib, and Cetuximab in BRAF V600E-Mutated Colorectal Cancer. N Engl J Med. 2019;381:1632–43. 10.1056/NEJMoa1908075 31566309

[45] Bridgewater JA, Pugh SA, Maishman T, Eminton Z, Mellor J, Whitehead A (2020). Systemic chemotherapy with or without cetuximab in patients with resectable colorectal liver metastasis (New EPOC): long-term results of a multicentre, randomised, controlled, phase 3 trial. Lancet Oncol.

[46] Primrose J, Falk S, Finch-Jones M, Valle J, O’Reilly D, Siriwardena A (2014). Systemic chemotherapy with or without cetuximab in patients with resectable colorectal liver metastasis: the New EPOC randomised controlled trial. Lancet Oncol..

[47] Maurer CA, Friess H, Kretschmann B, Zimmermann A, Stauffer A, Baer HU (1998). Increased expression of erbB3 in colorectal cancer is associated with concomitant increase in the level of erbB2. Hum. Pathol..

[48] Wullweber A, Strick R, Lange F, Sikic D, Taubert H, Wach S, et al. Bladder tumor subtype commitment occurs in carcinoma in-situ driven by key signaling pathways including ECM remodeling. Cancer Res. 2021;81:1552–66. 10.1158/0008-5472.CAN-20-2336 33472889

[49] Li M, Liu F, Zhang F, Zhou W, Jiang X, Yang Y, et al. Genomic ERBB2/ERBB3 mutations promote PD-L1-mediated immune escape in gallbladder cancer: a whole-exome sequencing analysis. Gut. 2018;68:1024–33. 10.1136/gutjnl-2018-316039 29954840

[50] Sergina NV, Rausch M, Wang D, Blair J, Hann B, Shokat KM (2007). Escape from HER-family tyrosine kinase inhibitor therapy by the kinase-inactive HER3. Nature..

[51] Nakata S, Tanaka H, Ito Y, Hara M, Fujita M, Kondo E (2014). Deficient HER3 expression in poorly-differentiated colorectal cancer cells enhances gefitinib sensitivity. Int J Oncol.

[52] Wilson FH, Politi K (2018). ERBB Signaling Interrupted: Targeting Ligand-Induced Pathway Activation. Cancer Discov..

[53] Barber PR, Weitsman G, Lawler K, Barrett J, Rowley M, Rodriguez-Justo M, et al. HER2-HER3 heterodimer quantification by FRET-FLIM and patient subclass analysis of the COIN colorectal trial. J Natl Cancer Inst. 20191;12:944–54. 10.1093/jnci/djz231 PMC7492762 31851321

[54] Reddy TP, Choi DS, Anselme AC, Qian W, Chen W, Lantto J (2020). Simultaneous targeting of HER family pro-survival signaling with Pan-HER antibody mixture is highly effective in TNBC: a preclinical trial with PDXs. Breast Cancer Res..

[55] Kiavue N, Cabel L, Melaabi S, Bataillon G, Callens C, Lerebours F, et al. ERBB3 mutations in cancer: biological aspects, prevalence and therapeutics. Oncogene. 2019;39:487–502. 10.1038/s41388-019-1001-5 31519989

[56] Fiori LM, Kos A, Lin R, Théroux JF, Lopez JP, Kühne C, et al. miR-323a regulates ERBB4 and is involved in depression. Mol Psychiatry. 2020;26:4191–204. 10.1038/s41380-020-00953-7 33219358

[57] Xu XH, Song W, Li JH, Huang ZQ, Liu YF, Bao Q (2021). Long Non-coding RNA EBLN3P Regulates UHMK1 Expression by Sponging miR-323a-3p and Promotes Colorectal Cancer Progression. Front Med.

[58] Shi P, Zhang J, Li X, Li W, Li H, Fu P. Long non-coding RNA NORAD inhibition upregulates microRNA-323a-3p to suppress tumorigenesis and development of breast cancer through the PUM1/eIF2 axis. Cell Cycle. 2021;20:1–13. 10.1080/15384101.2021.1934627 PMC8331030 34125645

