# Supplementary material for: MiR-323a regulates ErbB3/EGFR and blocks gefitinib resistance acquisition in colorectal cancer
Source: Cell Death Dis. 2022 Mar 22;13(3):256. doi: 10.1038/s41419-022-04709-9 (PMC8940899; doi:10.1038/s41419-022-04709-9)
Supplement: Supplementary file 1 — SUPPLEMENTAL MATERIAL [file 41419_2022_4709_MOESM1_ESM.docx]

Supplementary Figure 1


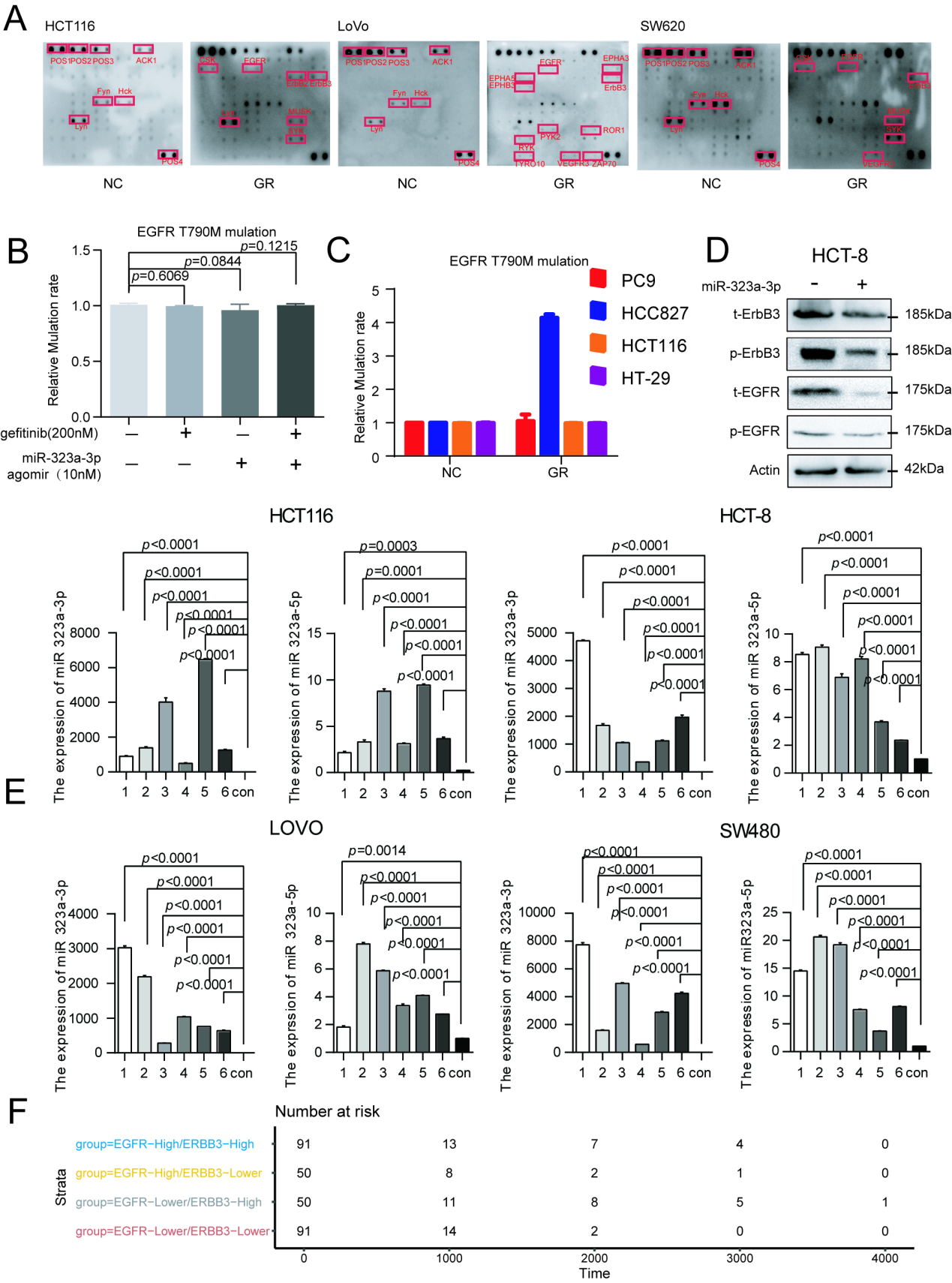


Supplementary Figure 1. Construction of gefitinib-resistant cell lines, establishment of miR-323a-3p over-expressing cell lines, and detection the RTK phosphorylation level in drug-resistant cell lines. A. Raw data of RTK phosphorylation level in HCT116, LoVo, and SW620. B. There is no mutation in EGFR T790M in MiR-323a-3p combined with gefitinib treatment subcutaneous tumor tissues. C. There was no EGFR T790M mutation in gefitinib-resistant CRC cell lines, and HCC827 was a positive control. D. MiR-323a-3p inhibited the protein level of EGFR/ErbB3 in the HCT-8 cell line. E. Construction of four stable over-expression 323 cell lines (HCT116, HCT-8, LoVo, and SW480). Levels of miR-323a-3p following miRNA overexpression were evaluated in HCT116, HCT-8, LoVo, and SW480 monoclonal cells. F. The number of patients for each subset in survival analysis (TCGA datasets).

Supplementary Figure 2


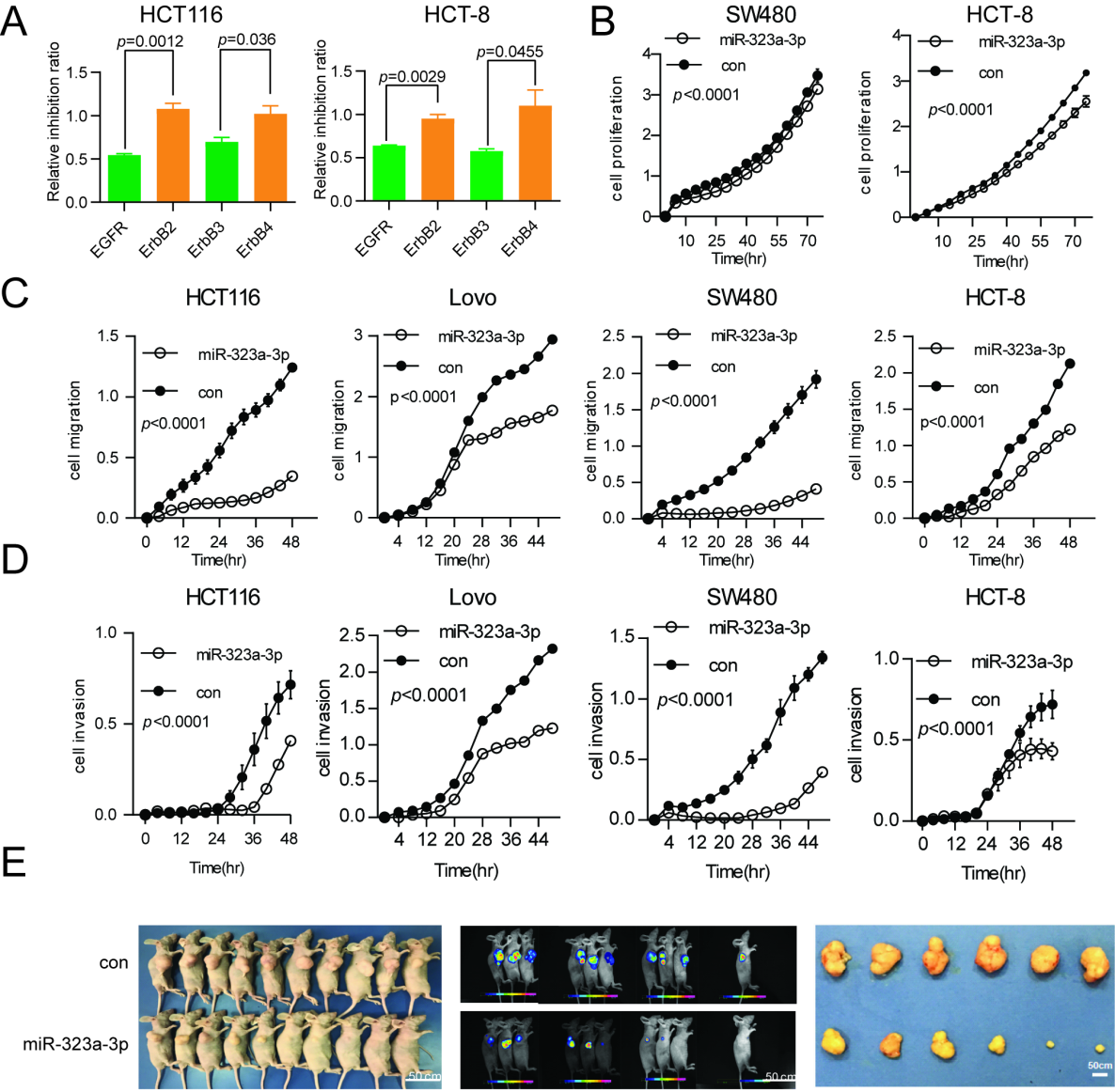


Supplementary Figure 2. MiR-323a-3p inhibits tumor growth by targeting EGFR/ERBB3. A. MiR-323a-3p inhibited RNA levels of EGFR/ ErbB3 in HCT116 and HCT-8. B. MiR-323a-3p inhibited the proliferation in SW480 and HCT-8 cells. C. MiR-323a-3p inhibited the migration in HCT116, HCT-8, LOVO, and SW480 cell lines. D. MiR-323a-3p inhibited the invasion in HCT116, HCT-8, LOVO, and SW480 cell lines. E. MiR-323a-3p inhibited the growth in HCT116 subcutaneous tumor model.

Supplementary Figure 3


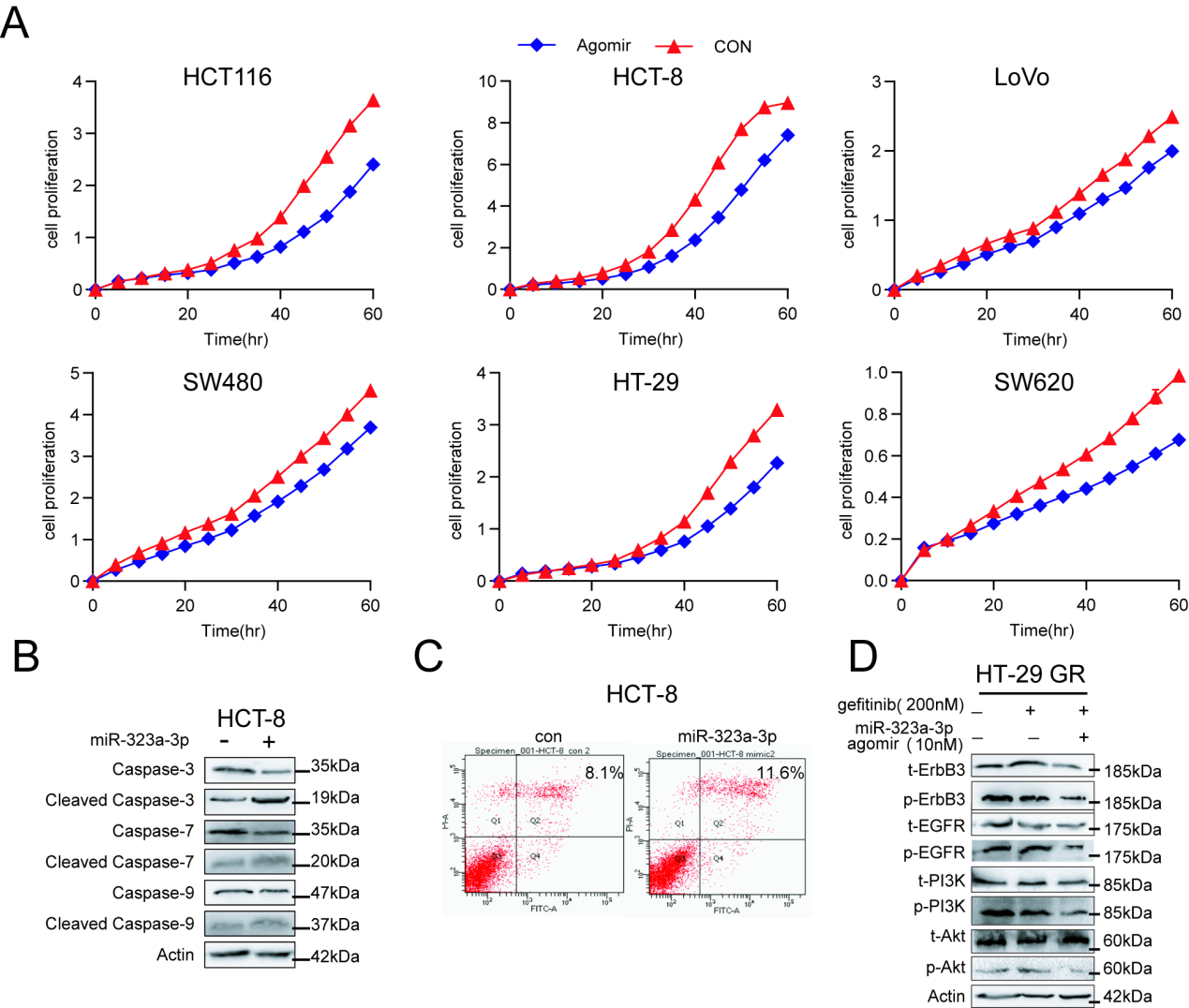


Supplementary Figure 3. MiR-323a-3p promotes apoptosis by inhibiting activation of EGFR/ ErbB3-PI3K /Akt. A. MiR-323a-3p inhibits cell proliferation in HCT116, HCT-8, LOVO, HT-29, SW620, and SW480. B. MiR-323a-3p promoted the rate of apoptosis in HCT-8 cell line. C. MiR-323a-3p promotes apoptosis marker activation in HCT-8 cell line. D. Phosphorylated EGFR, ErbB3, PI3K and Akt proteins in HT-29 GR cells did not change after gefitinib administration, but they were significantly reduced after the addition of agomir (n=3 per group).

Supplementary Figure 4


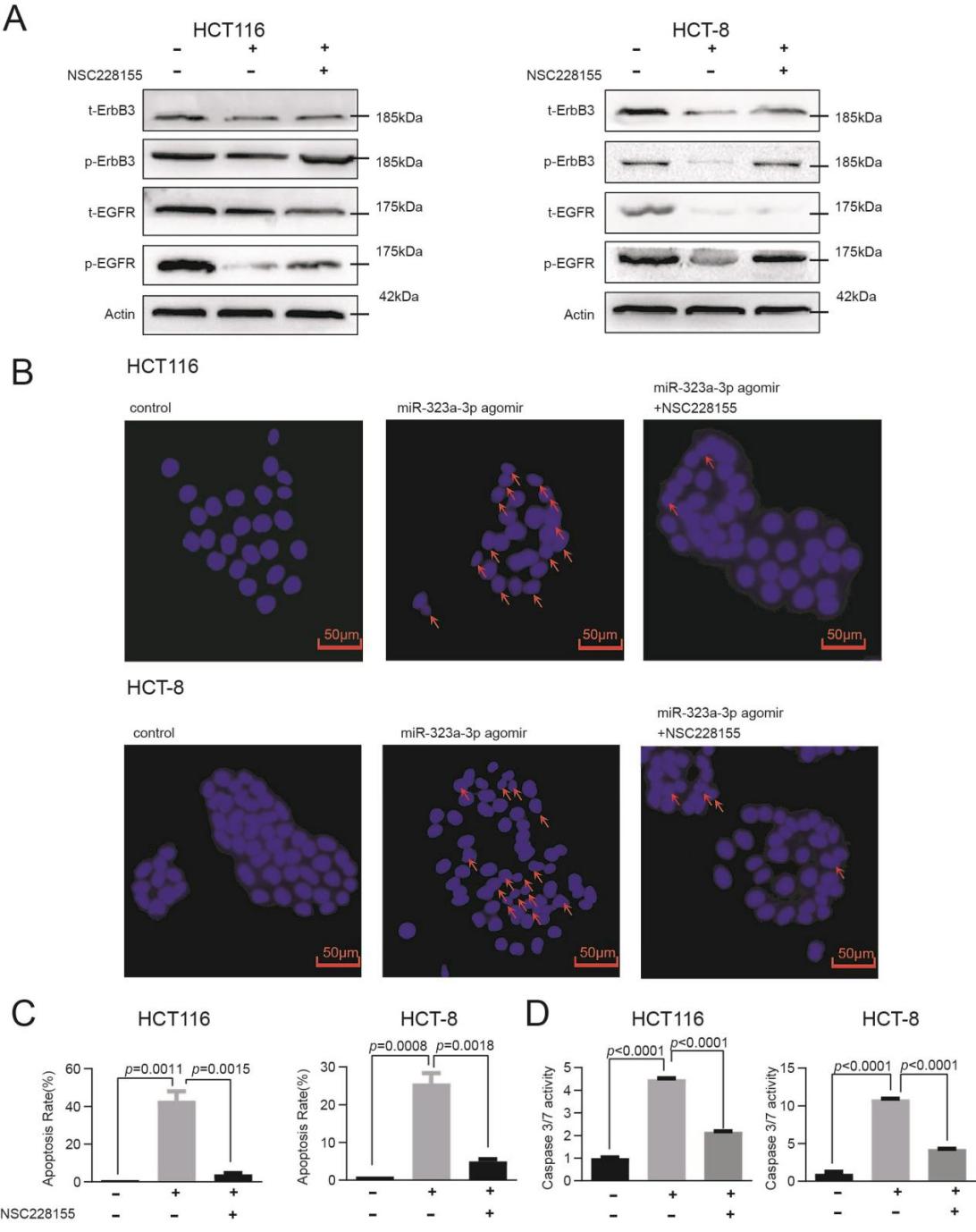


Supplementary Figure 4. Rescue experiment of miR-323a-3p targeting EGFR/ErbB3. A. Phosphorylated protein expression levels of EGFR and ErbB3 in HCT116 and HCT-8 were significantly increased by NSC228155 (EGFR agonist). The pro-apoptotic effect of agomir was counteracted by NSC228155. B. Hoechst 33258 fluorescent dye in HCT116 and HCT-8 were decreased by NSC228155. C. Analysis of the Hoechst 33258 fluorescent dye apoptosis rate. D. Caspase3/7 activity in HCT116 and HCT-8 were decreased by NSC228155.

Supplementary Figure 5


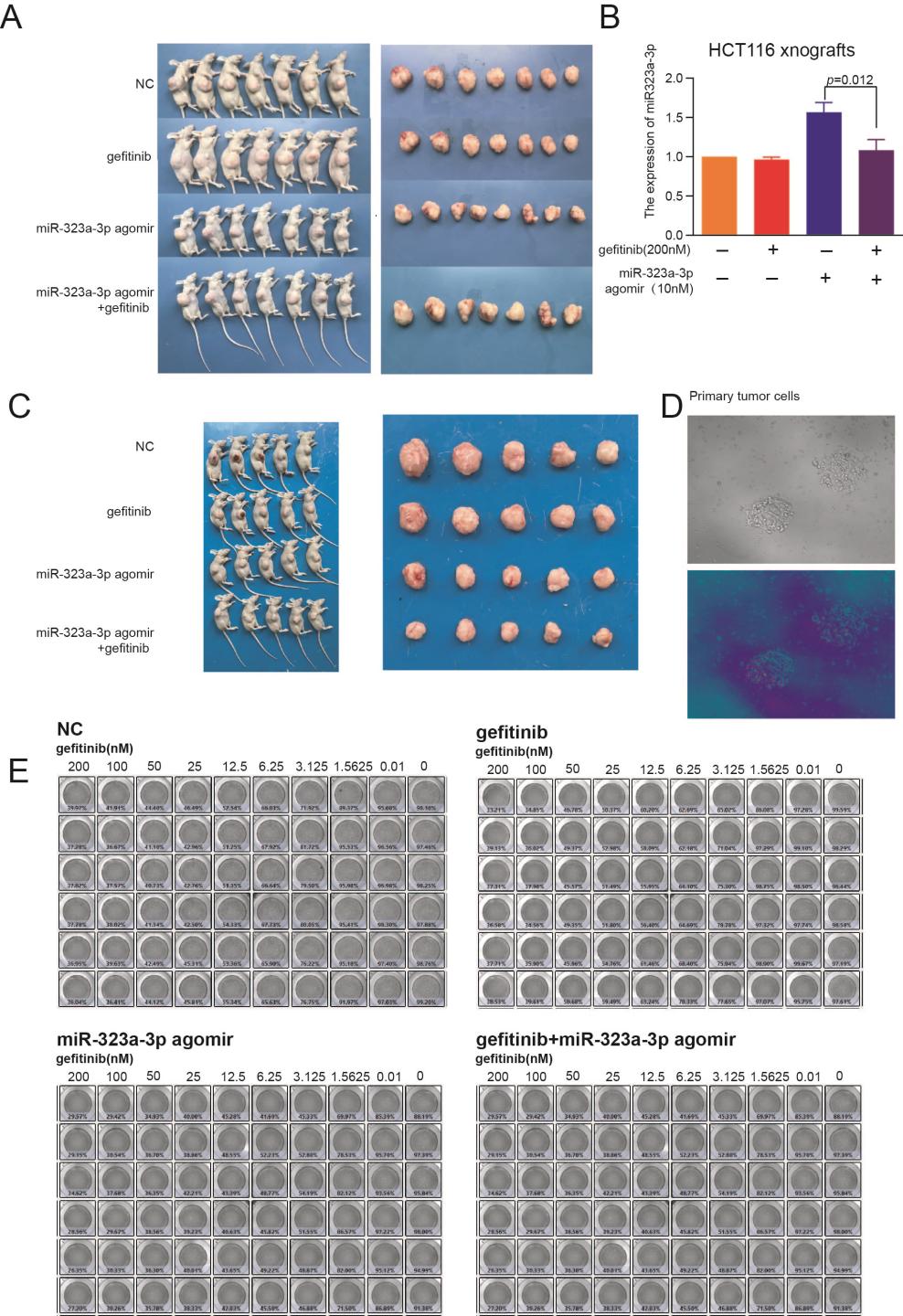


Supplementary Figure 5. MiR-323a-3p and gefitinib synergistically inhibit tumor growth, and miR-323a-3p blocks acquired gefitinib resistance formation in a xenograft model. A. The volume of subcutaneous tumors decreased more significantly with the combination of gefitinib and agomir administration than with single administration. B.The miR-323a-3p level was up-regulated in the miR-323a-3p agomiR treated subcutaneous tumors model. C. Subcutaneous tumors grew more slowly and survived longer with the combination of gefitinib and agomir than with single dosing of either agent. D. Construction of primary tumor cells. E. The IC50 of gefitinib in tumor progenitor cells in the xenograft model was much lower in the coadministered group than in the single agent-administered group.

**Supplementary Figure 6**


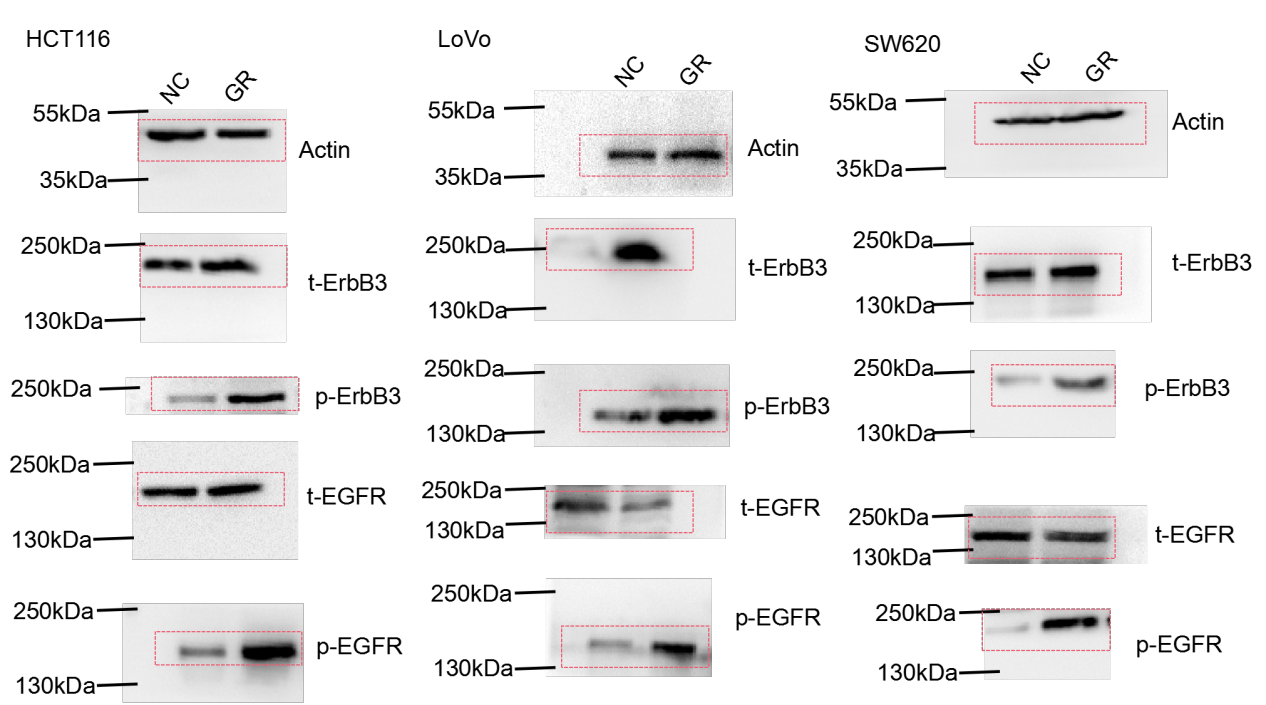


Supplementary Figure 6. The unedited western blot images, corresponding to the gel images in Figure 1D.

**Supplementary Figure 7**


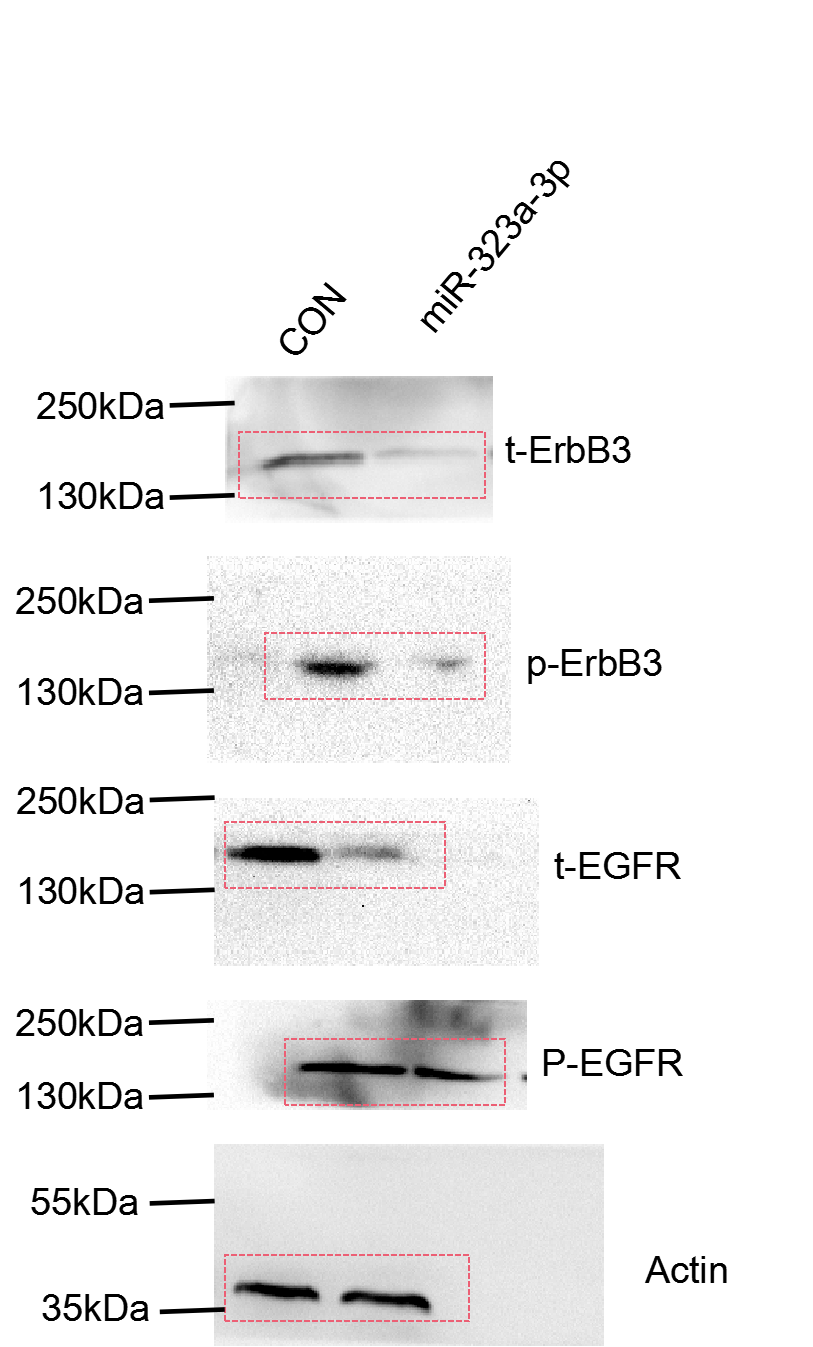


Supplementary Figure 7. The unedited western blot images, corresponding to the gel images in Figure 2C.

**Supplementary Figure 8**


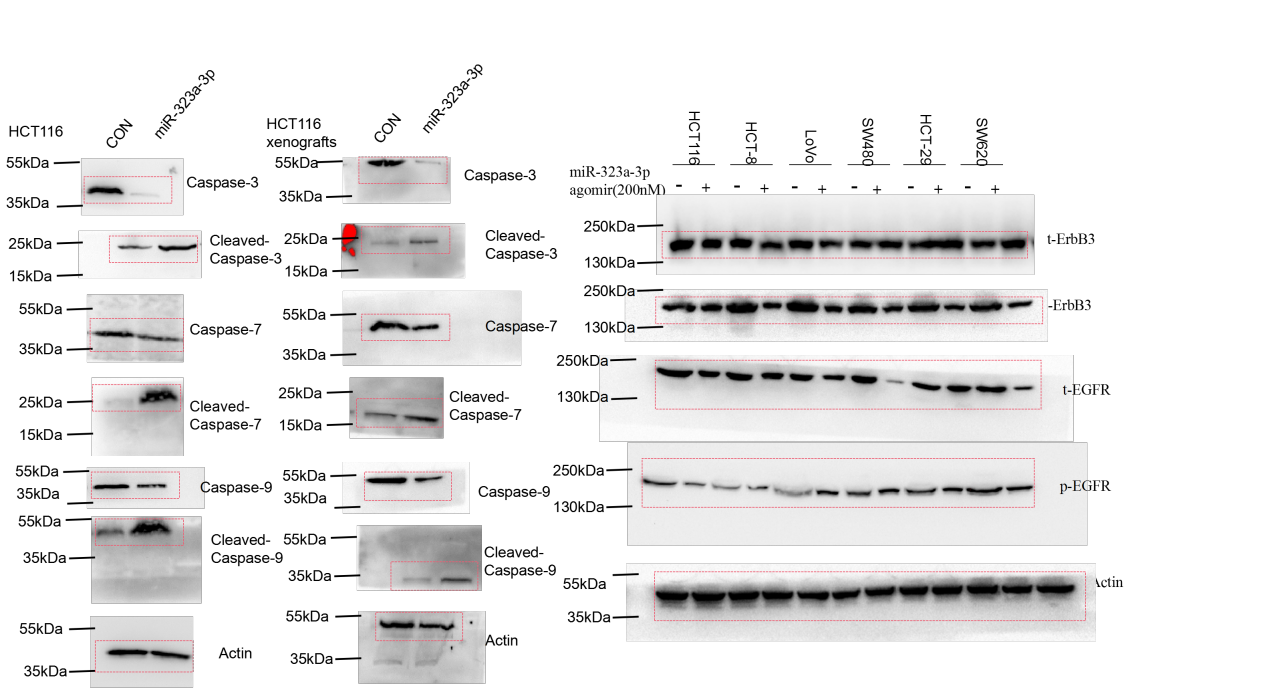


Supplementary Figure 8. The unedited western blot images, corresponding to the gel images in Figure 3B, 3C and 3G.

**Supplementary Figure 9**


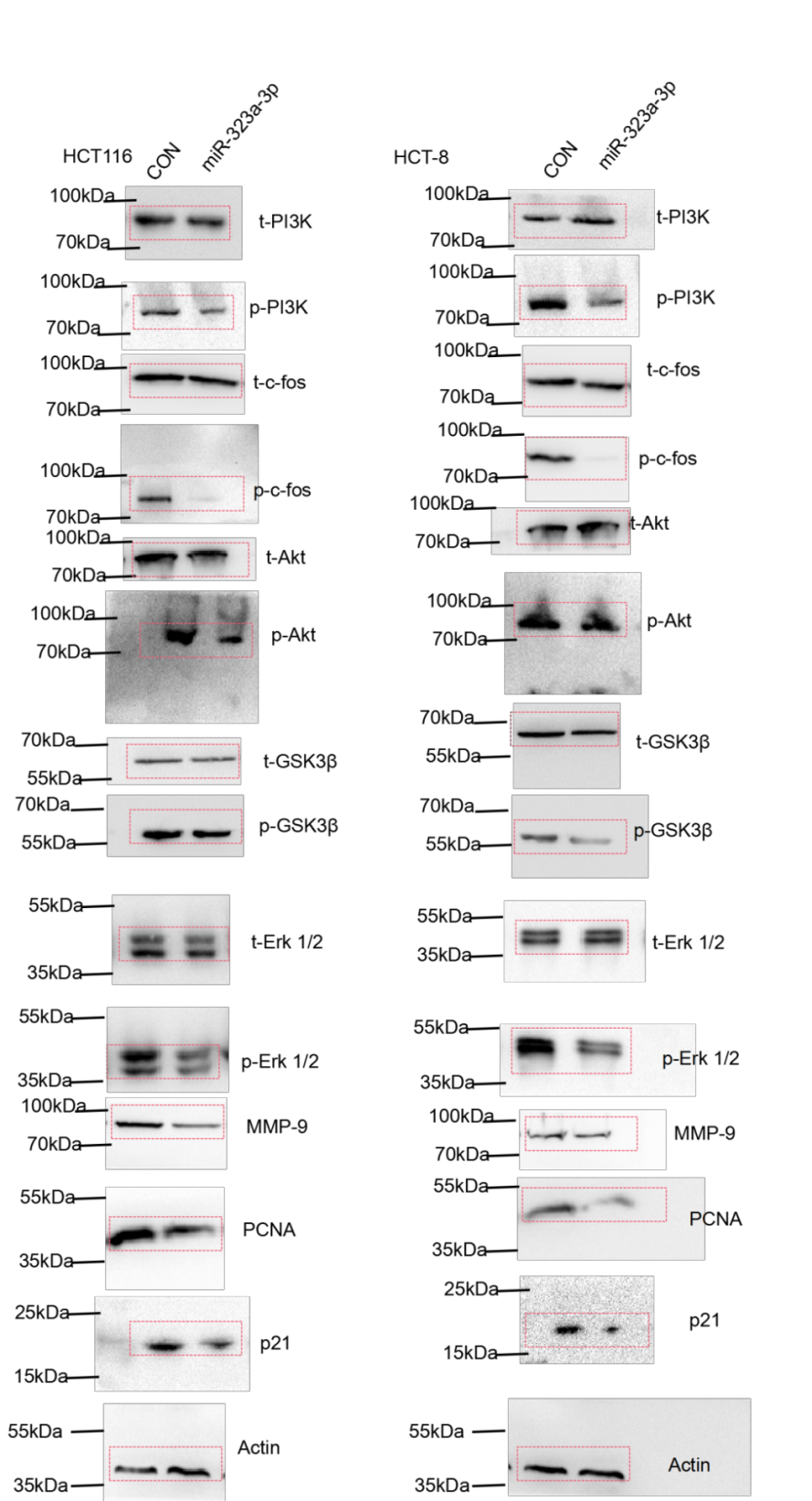


Supplementary Figure 9. The unedited western blot images, corresponding to the gel images in Figure 4D.

**Supplementary Figure 10**


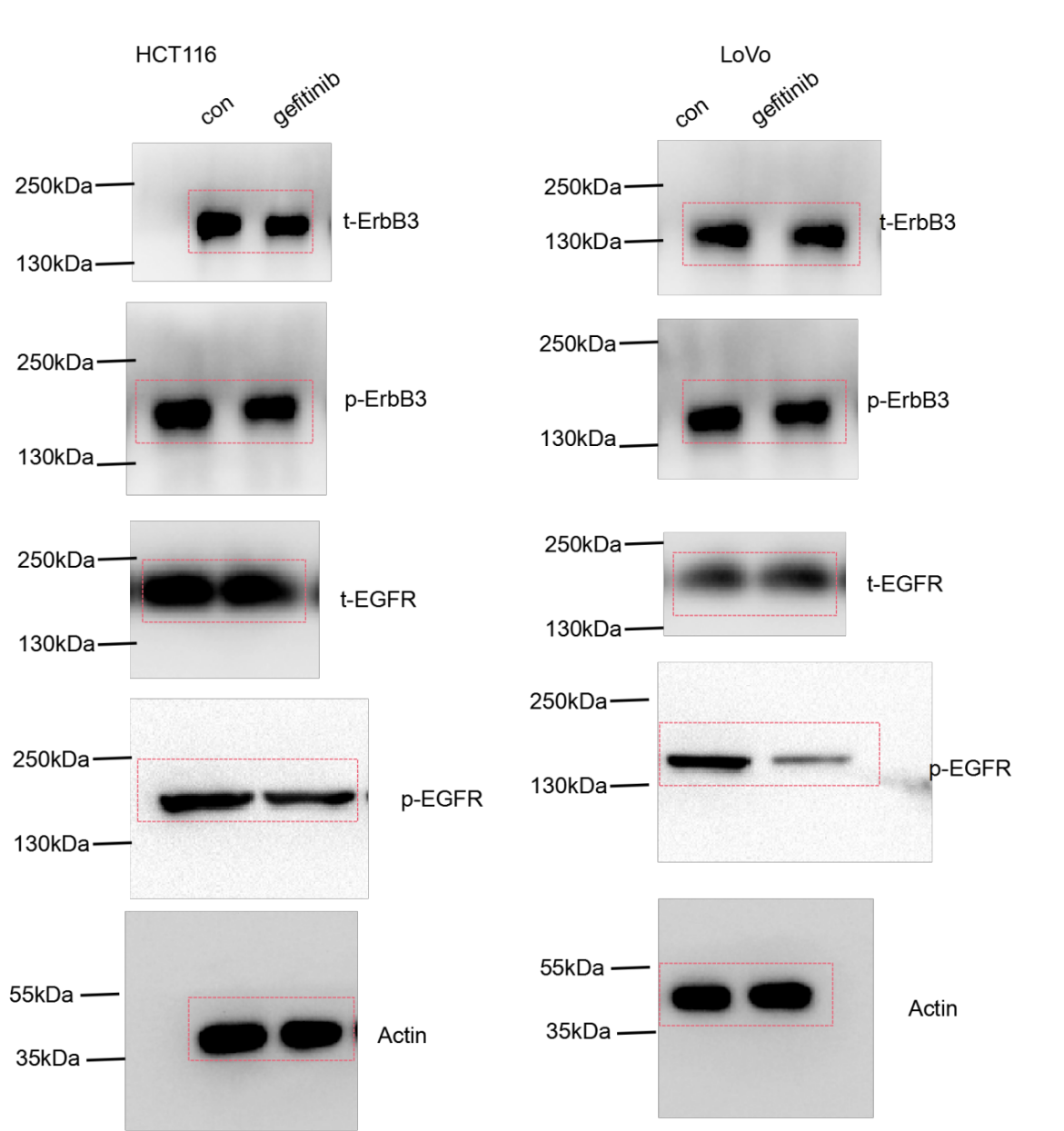


Supplementary Figure 10. The unedited western blot images, corresponding to the gel images in Figure 5A.

**Supplementary Figure 11**


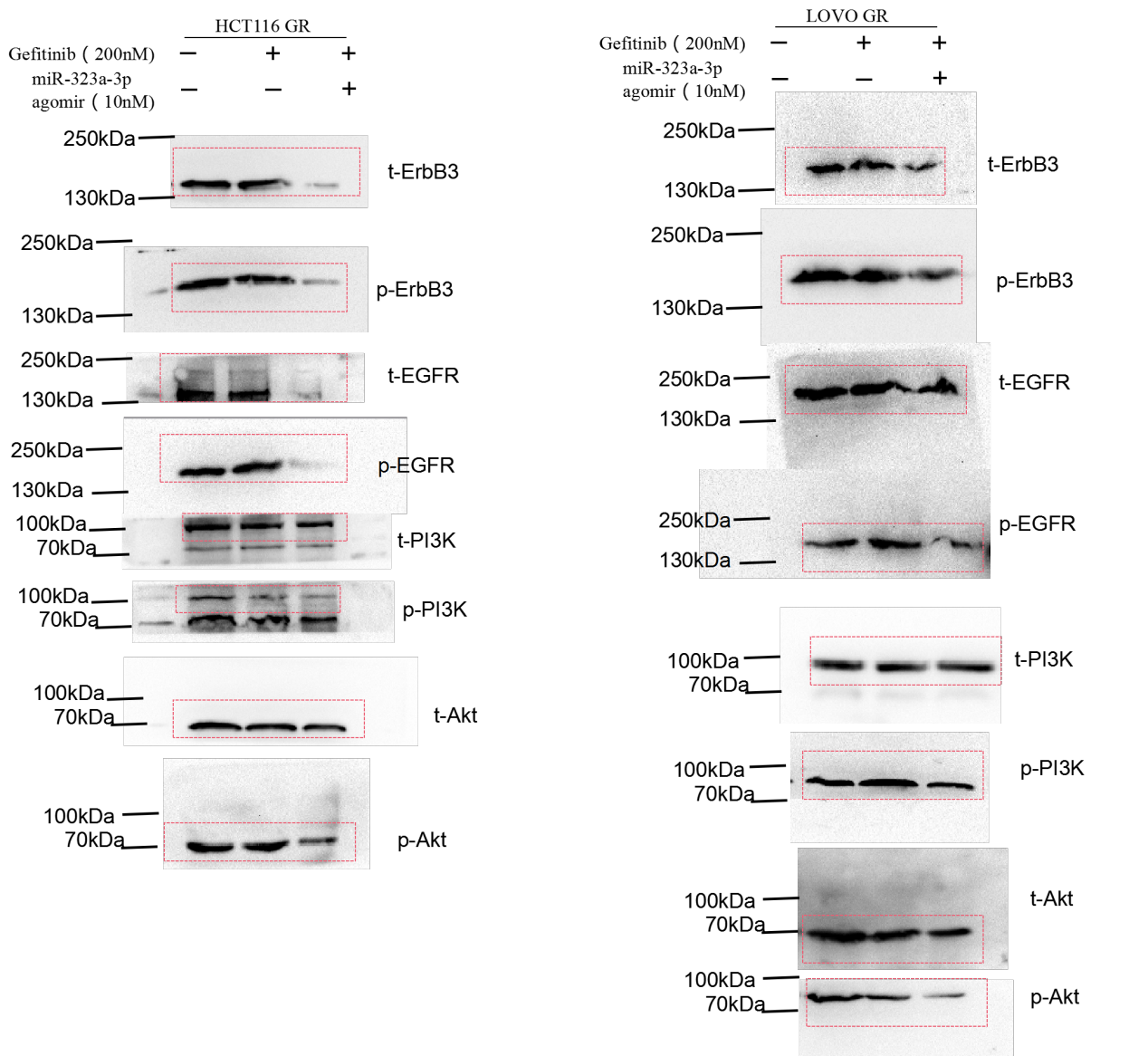


Supplementary Figure 11. The unedited western blot images, corresponding to the gel images in Figure 5B.

**Supplementary Figure 12**


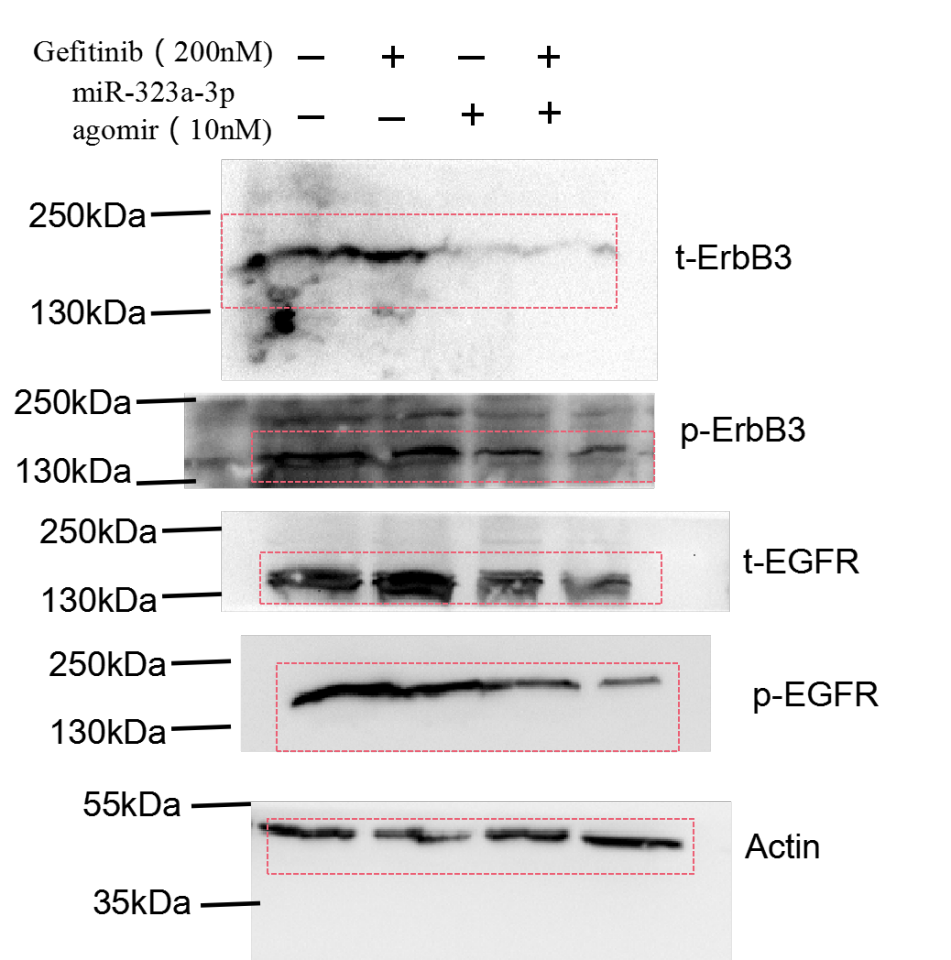


Supplementary Figure 12. The unedited western blot images, corresponding to the gel images in Figure 6E.
